# In Silico and In Vitro Evaluation of δ-cadinene from *Decatropis bicolor* as a Selective Inhibitor of Human Cell Adhesion and Invasion Proteins

**DOI:** 10.3390/cancers17172839

**Published:** 2025-08-29

**Authors:** Iannel Reyes-Vidal, Ivan Tepale-Ledo, Gildardo Rivera, Emma Ortiz-Islas, Salvador Pérez-Mora, David Guillermo Pérez-Ishiwara, Yazmin Montserrat Flores-Martinez, Maricarmen Lara-Rodríguez, María del Consuelo Gómez-García

**Affiliations:** 1 Laboratorio de Biomedicina Molecular I, Programa de Doctorado en Ciencias en Biotecnología, Escuela Nacional de Medicina y Homeopatía (ENMyH), Instituto Politécnico Nacional, Mexico City 07320, Mexico; ireyesv1917@alumno.ipn.mx (I.R.-V.); itepalel1900@alumno.ipn.mx (I.T.-L.); sperezm1510@alumno.ipn.mx (S.P.-M.); dperez@ipn.mx (D.G.P.-I.); yfloresma@ipn.mx (Y.M.F.-M.); mlarar2300@alumno.ipn.mx (M.L.-R.); 2 Laboratorio de Biotecnología Farmacéutica, Centro de Biotecnología Genómica, Instituto Politécnico Nacional, Reynosa 88710, Mexico; giriveras@ipn.mx; 3 Laboratorio de Neurofarmacología Molecular y Nanotecnología, Instituto Nacional de Neurología y Neurocirugía “Manuel Velasco Suárez”, Mexico City 14269, Mexico; emma.ortiz@innn.edu.mx

**Keywords:** breast cancer, extracellular matrix, terpenoids, molecular docking, sesquiterpene, MMP-2, adhesion proteins, *Decatropis bicolor*, δ-cadinene

## Abstract

**Background**: Breast cancer is a complex, multifactorial malignancy characterized by the uncontrolled proliferation of epithelial cells, with certain subtypes exhibiting resistance to conventional therapies. Plant-derived essential oils have been proposed as potential anticancer agents due to their bioactive compounds. Recent studies have demonstrated that *Decatropis bicolor* essential oil exhibits activity against breast cancer, attributed to diverse secondary metabolites such as δ-cadinene. Aberrant expression of adhesion and invasion proteins, including MMPs, CD44, N-cadherin, and ZEB-2, are key signs of breast cancer progression and metastasis; they represent relevant molecular targets. **Objectives**: To investigate the interaction of δ-cadinene with these proteins using in silico approaches and in vitro evaluations. **Methods**: In silico analyses were conducted to assess the interaction and stability of δ-cadinene with target proteins. In vitro assays, including cytotoxicity, morphological analysis, and cell invasion assays, were performed using MDA-MB-231 and MCF10-A cell lines. **Results**: Interaction analysis suggest that δ-cadinene interacts with key catalytic residues in MMP-2, sharing features with Quercetin. Blind docking revealed a second high-affinity site in the Fibronectin type II domain. Molecular dynamics simulations confirmed the stability of these complexes. In vitro studies showed that δ-cadinene significantly reduced MDA-MB-231 cell viability in a concentration-dependent manner, without affecting MCF10-A cells, and significantly inhibited invasion and MMP-2 activity after 24 h. **Conclusions**: δ-cadinene exhibits selective cytotoxic and anti-invasive activity in MDA-MB-231 cells, likely through dual inhibition of the catalytic and adhesion domains of MMP-2. These findings support δ-cadinene as a potential candidate for future therapeutic development in metastatic breast cancer.

## Simple Summary

Breast cancer remains an aggressive disease with limited therapeutic alternatives. In this study, we investigated the anticancer effects of δ-cadinene on the MDA-MB-231 breast cancer cell line. δ-cadinene significantly reduced cell viability, impaired invasive capacity, and altered cell morphology, while exerting minimal effects on normal MCF10-A cells. Computational approaches, including molecular docking and molecular dynamics simulations, revealed a strong and stable interaction between δ-cadinene and MMP-2, a key protein involved in cancer invasion. These findings suggest that δ-cadinene is a promising candidate for targeted breast cancer therapy modulating MMP-2.

## 1. Introduction

Breast cancer (BC) is a heterogeneous neoplasm that originates from various epithelial cells of the mammary gland [1]. Globally, an estimated 1.7 million cases of BC occur among women aged 15–50 years. BC remains a major public health concern with a survival rate of 5 years [2]. A key characteristic of BC is its high capacity to metastasize to various organs and tissues, a process regulated by cell migration and invasion mechanisms that facilitate disease progression [3]. Initially, tumor cells undergo epithelial–mesenchymal transition (EMT), characterized by loss of the epithelial phenotype and acquisition of mesenchymal phenotype, including loss of apical–basal polarity, cell junctions, and cytoskeletal organization. This transition enhances motility and enables intravasation into the systemic circulation [4]. Moreover, metastatic dissemination is the leading cause of cancer-related mortality largely driven by extracellular matrix (ECM) remodeling and altered cell adhesion dynamics in BC. Consistent evidence across multiple tumor types, including BC, underscores the biological relevance of adhesion and invasion molecules and highlights their potential as both prognostic biomarkers and therapeutic targets [5]. Matrix metalloproteinases (MMPs), particularly MMP-2 and MMP-9, are Zn^2+^-dependent endopeptidases that degrade ECM structural components, facilitating tumor dissemination and invasion [6]. N-cadherin and the transcription factor ZEB-2 are central EMT effectors that promote metastatic progression in BC [6,7,8]. CD44, a versatile adhesion receptor and cancer stem cell marker, is frequently overexpressed in triple-negative breast cancer (TNBC). It mediates cell–matrix interactions and activates downstream signaling pathways that enhance survival, migration, and therapy resistance [9]. This dual role, acting as both prognostic biomarkers and therapeutic targets, highlights their importance in tumor progression and underscores their potential for developing novel therapeutic strategies. Multimodal therapy for BC includes surgery, chemotherapy, radiotherapy, and hormone therapy [10,11]. These therapeutic strategies for BC are often associated with significant side effects, underscoring the need for novel molecules that minimize toxicity while providing targeted anti-tumor effects. Plant-derived essential oils (EOs) consist of volatile secondary metabolites, primarily terpenes, which play a pivotal role in plant defense, antimicrobial activity, and have been used in medicine to treat various diseases since ancient times [12]. Recent research highlights their potential as anticancer agents, revealing mechanisms of action, such as the induction of apoptosis, inhibition of proliferation, and modulation of signaling pathways involved in cell adhesion and invasion [13]. Various studies have demonstrated the anticancer activity of EOs and their main components in cell lines and animal cancer models [13].

However, the medical use of these EOs in medicine has been limited due to their highly volatile and hydrophobic chemical properties and because they contain numerous compounds that may induce adverse effects [14]. Plant-derived EOs contain a wide range of secondary metabolites, which, despite showing promising therapeutic potential, often present limitations that hinder their development as therapeutic agents. Among these limitations, low water solubility results in poor absorption and, consequently, reduced bioavailability, which may require higher concentrations to achieve a significant therapeutic effect [15]. This condition complicates not only the design of stable formulations but also their administration. Additionally, the limited information available on their pharmacokinetic characteristics makes it difficult to fully understand their disposition within the body [16]. Therefore, identifying and characterizing the metabolites responsible for these anticancer effects is imperative. In silico analysis and bioinformatics have facilitated advances in characterizing metabolites with potential anticancer effects potentiating the identification of highly promising candidates [16]. For example, the essential oil (EO) of *Artemisia sieberi* exhibited pronounced cytotoxic activity against the MCF-7 BC cell line. Furthermore, it suppressed cell migration, induced cell cycle arrest in the S phase, and promoted apoptosis in the MCF-7 cell line (IC_50_ value of 38.7 μg/mL) by downregulating of the ERK signaling pathway [17].

Similarly, the EO of *Erythrina corallodendron* exhibited significant anticancer activity by inhibiting the proliferation, migration, and invasion of MCF-7 and MDA-MB-231 BC cell lines, with IC_50_ values of 4.9 ± 0.3 μg/mL and 3.4 ± 0.2 μg/mL, respectively [18]. Notably, it induced a morphological transition in cancer cells, shifting from a loose, spindle-shaped phenotype to a tight, rounded morphology, indicative of a reversal of the EMT. This effect was corroborated by the downregulation of key EMT markers, including N-cadherin, Vimentin, Snail, and Slug, at both the mRNA and protein levels [18]. Recently, the EO of *Decatropis bicolor (D. bicolor)*, a shrub native to central Mexico, has demonstrated specific cytotoxic and apoptotic effects in the BC cell line MDA-MB-231 (IC_50_ = 53.9  ±  1.7 μg/mL) [19]. Our group observed that a subfraction of fraction II of *D. bicolor* EO significantly reduced the viability of MDA-MB-231 cells in a concentration-dependent manner, while exhibiting no cytotoxic effect on non-tumorigenic MCF10-A cells [20]. Furthermore, δ-cadinene (a phytochemical of that subfraction) showed the highest relative abundance, suggesting that this sesquiterpene might be responsible for the antitumor effect in BC cells. δ-cadinene, induces significant morphological changes in human ovarian cancer cells (OVCAR-3) and exhibits concentration- and time-dependent growth inhibitory effects [21]. Apoptotic features including cell shrinkage, chromatin condensation, and nuclear membrane rupture were observed in OVCAR-3 cells following treatment with δ-cadinene. Additionally, δ-cadinene induced a concentration-dependent cell cycle arrest in the sub-G1 phase [21]. Our current study suggests that δ-cadinene acts as a promising selective inhibitor of key proteins involved in cellular adhesion and invasion, supporting its potential as a therapeutic candidate in BC.

## 2. Materials and Methods

### 2.1. Preparation of δ-cadinene Ligand

The 2D structure and SMILES code of δ-cadinene were retrieved from the PubChem database (https://pubchem.ncbi.nlm.nih.gov/, accessed on 20 October 2024; PubChem CID: 441005). Ligand preparation involved charge minimization using the Gasteiger method, followed by optimization to a low-energy conformation with Open Babel [22]. The optimized structure was then converted into a 3D format and saved as an .SDF file for subsequent docking analyses.

### 2.2. Crystallographic Structures of MMP-9, CD44 and ZEB-2

The crystal structure of the catalytic domain of human MMP-9, determined by X-ray diffraction at 1.30 Å resolution (PDB ID: 4XCT), was used in this study. Similarly, the crystal structure of the hyaluronic acid–binding domain of human CD44 was obtained by X-ray diffraction at 2.20 Å resolution (PDB ID: 1UUH). Additionally, the solution structure of the homeobox domain of the zinc finger homeobox protein 1b (Smad-interacting protein 1) was retrieved (PDB ID: 2DA7). All 3D structures were downloaded from the Protein Data Bank (PDB; https://www.rcsb.org/, accessed on 9 November 2024) [23].

### 2.3. Acquisition and Validation of 3D Structural Models for Human MMP-2 and N-cadherin

Because complete X-ray crystallographic structures for *Homo sapiens* MMP-2 and N-cadherin are not available, we used models from the AlphaFold Protein Structure Database (AF-DB, EMBL-EBI; CC BY 4.0) [24]. Protein sequences were obtained from UniProt (https://www.uniprot.org; accessed on 31 October 2024): MMP-2 (UniProt: P08253, 660 aa) and N-cadherin (UniProt: P19022, 906 aa). The corresponding AF-DB entries (https://alphafold.ebi.ac.uk/entry/P08253 and https://alphafold.ebi.ac.uk/entry/P19022; accessed on 31 October 2024) were downloaded in .pdb format and used for downstream analyses. Predicted confidence values (pLDDT) were obtained directly from the AF-DB pages for each model. To account for the essential role of Zn^2+^ in the catalytic activity of MMP-2, we constructed a Zn^2+^-bound model of the human protein. Metal ion coordination was predicted and modeled using the MIB2: Metal Ion-Binding Site Prediction and Modeling Server [25] (https://combio.life.nctu.edu.tw/MIB2/; accessed on 5 August 2025), which integrates sequence and structural features to identify putative metal-binding sites. This approach not only allowed us to incorporate the catalytic Zn^2+^ ion and ensure a physiologically relevant conformation but also provided a reference model for direct comparison with MD results obtained in the absence of the ion. The models were validated by assessing their stereochemical quality and structural integrity using well-established computational tools. First, PDBsum (version 2024; https://www.ebi.ac.uk/thornton-srv/databases/pdbsum/Generate.html, accessed on 9 November 2024) [26] was employed to analyze the phi (φ) and psi (ψ) torsion angles through Ramachandran plots, providing detailed insights into the global stereochemical conformation. Second, ProSA-web (version 2007; https://prosa.services.came.sbg.ac.at/prosa.php, accessed on 10 November 2024) [27] was used to calculate Z-scores, which were benchmarked against experimentally determined X-ray crystallographic structures, thereby assessing overall model quality and energy profiles. Finally, ERRAT (version 6; https://saves.mbi.ucla.edu/, accessed on 10 November 2024) [28] was applied to evaluate non-bonded atomic interactions and residue-specific environments within the 3D models.

### 2.4. Receptor Preparation and Molecular Docking Simulation

For the MD analysis of MMP-9, the X-ray crystal structure with a resolution of 1.30 Å (PDB ID: 4XCT) was selected. For CD44, the crystal structure with a resolution of 2.20 Å resolution (PDB ID: 1UUH) was used. The NMR solution structure of the homeobox domain of zinc finger homeobox protein 1b (ZEB-2) is available as an ensemble of 20 models (PDB ID: 2DA7). Model 7, corresponding to the medoid conformation, was chosen as the representative structure because it displayed the lowest average root mean square deviation (RMSD) relative to all other models, thereby ensuring the most statistically representative conformation for subsequent in silico analyses.

Prior to docking, all water molecules, ions, ligands, and cofactors were removed from the protein structures using UCSF Chimera (version 1.10.2) [29] to prevent potential interference with binding site accessibility. DockPrep (version 1.17.3) was employed to add polar hydrogens and repair incomplete side chains. Gasteiger charges were assigned using MGLTools (version 1.5.6), and the processed files were subsequently converted into PDBQT format [29]. The resulting docked complexes were visualized and analyzed using Discovery Studio 2024 (Dassault Systèmes BIOVIA) and PyMOL (version 3.1; https://www.pymol.org/, accessed on 11 November 2024) [30]. Intermolecular interaction analysis was performed using Discovery Studio, generating 2D diagrams that evaluate potential hydrogen bonds, hydrophobic interactions (alkyl and π–alkyl; where alkyl bonds represent Van der Waals interactions according to the Discovery Studio nomenclature), electrostatic interactions (both attractive and repulsive), aromatic interactions (including π–π stacked, π–π T-shaped, π–cation, and π–anion), water-mediated hydrogen bonds, and Van der Waals contacts [31].

### 2.5. Validation of Molecular Docking

The MD analyses were conducted using AutoDock 4 (version 4.2.6) [29] and AutoDock Vina (version 1.1.2) [32]. For non-blind docking, grids were centered on the known catalytic or ligand-binding sites of each target protein, with a consistent grid spacing of 0.375 Å applied across all analyses. AutoDock 4 docking parameters included the Lamarckian genetic algorithm with 50 runs, a population size of 150, a maximum of 2,500,000 energy evaluations, and up to 27,000 generations. For blind docking, larger grids encompassing the entire protein surface were employed, maintaining the same 0.375 Å spacing to enable unbiased exploration of potential binding sites. AutoDock Vina was executed with an exhaustiveness parameter of 9 for non-blind docking. The grid box dimensions, centers, and spacing were as follows: for N-cadherin in with ADH-1 (Exherin, PubChem CID: 9916058), 60 Å × 60 Å × 60 Å grid was centered at (10.288, 34.681, −41.554), corresponding to the protein’s catalytic site [33]. For CD44 with Mitoxantrone (PubChem CID: 4212), a 60 Å × 60 Å × 60 Å grid was constructed, centered at (0.032, −5.631, 24.284) within the catalytic site [34]. For ZEB-2 with Orientin (PubChem CID: 5281675), a 60 Å × 60 Å × 60 Å grid was defined, centered at (5.749, −21.116, 0.866) [35]. For MMP-9 with Quercetin (PubChem CID: 5280343), a 45 Å × 45 Å × 45 Å grid was used, centered at (20.683, −16.615, 19.006) [36]. Finally, for MMP-2 with Quercetin, a 65 Å × 65 Å × 65 Å a grid was defined, centered at (4.156, −12.044, 14.434) [37].

### 2.6. Molecular Dynamics Simulation

MDS were performed by selecting the most favorable binding pose for each compound from the MMP-2–ligand docking results. Topologies and parameters for the compounds with the highest binding affinities were generated using the Python interface (version 3.13) of Antechamber (ACPYPE) (https://www.bio2byte.be/acpype/, accessed on 20 December 2024) [38]. Protein topologies were prepared with GROMACS (version 2018.4) using the AMBER force field [39]. The complexes were solvated in a dodecahedral box using the TIP3P water model, maintaining a 10 Å buffer from the box edges, and neutralized with Na^+^ and Cl^−^ ions. Energy minimization was performed for 50,000 steps, followed by equilibration for 100 ps under both NVT (constant number of particles, volume, and temperature) and NPT (constant number of particles, pressure, and temperature) ensembles. Production simulations were then conducted at 300 K and 1 bar for 120 ns.

### 2.7. MDS Trajectory Analysis

We compared the atomic properties of the different complexes using the analysis tools included in GROMACS software (version 2018.4). Parameters such as the RMSD of the α-carbons and the ligand, the root mean square fluctuation (RMSF) of the α-carbons, the two-dimensional (2D) structure of MMP-2, and the radius of gyration (Rg) were evaluated to assess the stability of the complexes [39].

### 2.8. Energetic Contribution Analysis

At the end of the MDS, the ΔG_b_ values from 50 snapshots were calculated using molecular mechanics Poisson–Boltzmann surface area (MM-PBSA) methods for each complex with the g_mmpbsa program (version 2018.4) [40]. The interactions and their energetic contributions were analyzed from the last 10 ns of the MDS using the MmPbSaDecomp.py script.

### 2.9. Cell Culture and Reagents

The MDA-MB-231 cells were cultured in Dulbecco’s Modified Eagle’s Medium (DMEM; Gibco, Thermo Fisher Scientific, Waltham, MA, USA) supplemented with 10% fetal bovine serum (FBS; Thermo Fisher Scientific, Waltham, MA, USA) and 1% penicillin/streptomycin (Gibco, Thermo Fisher Scientific, Waltham, MA, USA). The MCF10-A cell line was cultured in DMEM/F12 medium supplemented with 10% FBS, 1 mg/mL hydrocortisone (Sigma-Aldrich, St. Louis, MO, USA), 100 µg/mL epidermal growth factor (Sigma-Aldrich, St. Louis, MO, USA), and 100 µg/mL insulin (Laboratorios Pisa, Guadalajara, Jalisco, Mexico). Both cell lines were maintained at 37 °C in a humidified atmosphere containing 5% CO_2_. δ-cadinene was obtained commercially from Finetech Industry Limited (Wuhan, Hubei, China) with a purity of 95% (Catalog Number: FT-0701082).

### 2.10. Treatments

MDA-MB-231 and MCF10-A cells (1 × 10^4^ cells/well) were seeded in a 96-well culture plates (Corning Inc., Corning, NY, USA) and incubated overnight. The cells were then treated with δ-cadinene at concentrations ranging from 0.3 to 30 µM for 24, 48, and 72 h. δ-cadinene was handled and stored under low-light conditions to prevent potential degradation due to light exposure. Concentration–response curves were generated to determine the half-maximal inhibitory concentration (IC_50_) of δ-cadinene. Paclitaxel (Sigma-Aldrich, St. Louis, MO, USA) was included as a positive control, based on a previously reported IC_50_ of 0.25 µg/mL. This concentration was converted to 0.3 µM using its molecular weight (853.91 g/mol) to allow direct comparison with the experimental compounds evaluated in micromolar units. This concentration has been shown to effectively inhibit proliferation in MDA-MB-231 cells under comparable conditions [19]. The 0.2% dimethyl sulfoxide (DMSO; Sigma-Aldrich, St. Louis, MO, USA) solvent control was also evaluated. Additionally, this study assessed the in vitro IC_50_ values of Quercetin (Sigma-Aldrich, St. Louis, MO, USA) (295 µM) [41] and 5-Fluorouracil (5-FU; Laboratorios Ulsatech, Guadalajara, Jalisco, Mexico) to evaluate their efficacy in inhibiting the proliferation, migration, and invasion of MDA-MB-231 cells. The concentrations of 5-FU used were selected based on previously reported IC_50_ values. Although the original data were expressed in µg/mL, they were converted to micromolar units using the molecular weight of 5-FU (130.08 g/mol) to maintain consistency with the other compounds evaluated in this study. The corresponding IC_50_ values were calculated as 265 µM at 24 h, 34.13 µM at 48 h, and 19.25 µM at 72 h [42].

### 2.11. Cytotoxicity Assay

Cell viability was assessed using a modified MTT assay (3-(4,5-dimethylthiazol-2-yl)-2,5-diphenyltetrazolium bromide; Sigma-Aldrich, St. Louis, MO, USA) following the method described by Mosmann [43].

Briefly, 10 µL of MTT reagent (5 mg/mL) dissolved in phosphate-buffered saline (PBS) was added to each well, and the cells were incubated for 4 h at 37 °C. The resulting formazan crystals were solubilized by adding 100 µL of DMSO, and absorbance was measured at 570 nm using a spectrophotometer. Results were expressed as the percentage of viable cells relative to the control group, calculated as follows:

Cell viability = (Control group Optical Density (OD)/Treatment group OD) × 100(1)

The experiment was performed in triplicate and repeated three times. The IC_50_ values and mean ± standard error of the mean (SEM) were calculated separately for each experiment using GraphPad Prism 8.0 software (GraphPad Software, Inc., Boston, MA, USA).

### 2.12. Predicted IC_50_ of δ-cadinene

The predicted sensitivity of δ-cadinene was assessed using the PaccMan platform (https://ibm.biz/paccmann-aas, accessed on 20 October 2024) [44]. The SMILES code corresponding to δ-cadinene was submitted to the model, which returned a theoretical IC_50_ value [log(µM)] across 2022 cancer cell lines included in the Genomics of Drug Sensitivity in Cancer (GDSC) and Cancer Cell Line Encyclopedia (CCLE) datasets. The theoretical IC_50_ value obtained for the MDA-MB-231 cell line was subsequently used to guide experimental validation assays, allowing direct comparison between predicted and observed cytotoxic responses.

### 2.13. Selective-Index

To evaluate the cytotoxic selectivity of the tested substances, the selectivity index (SI) was calculated using the following equation:

SI = (IC_50_ for normal cell line/IC_50_ for cancer cell line) (2)

According to Tronina et al. [45], SI values greater than 1.0 indicate a selective compound.

### 2.14. Cell Morphology Analysis

MDA-MB-231 and MCF10-A cells (9 × 10^4^) were seeded in an 8-well plate (Corning Inc., Corning, NY, USA). When the cell density reached approximately 80%, the cells were treated with the IC_50_ concentration of δ-cadinene for 24, 48, and 72 h. After incubation, the cells were washed twice with PBS and fixed in cold 4% paraformaldehyde solution (Sigma-Aldrich, St. Louis, MO, USA) for 1 h at 4 °C. The cells were then washed twice with PBS, stained with hematoxylin and eosin (H & E) (Sigma-Aldrich, St. Louis, MO, USA), and observed under an optical microscope equipped with a DP21 photographic system (Olympus, Tokyo, Japan) [19].

### 2.15. MMP-2 Enzyme Activity

MDA-MB-231 cells were cultured in serum-free DMEM medium with the IC_50_ of δ-cadinene at 24, 48, and 72 h. IC_50_ concentrations of Paclitaxel, Quercetin, and 5-FU were used as positive controls for cell damage. As negative controls, cells cultured with medium only and cells treated with 0.2% DMSO were included. Supernatants were collected, mixed with 5× non-reducing sample buffer (4% SDS, 20% glycerol, 0.01% bromophenol blue, and 125 mM Tris-HCl, pH 6.8), and analyzed to detect MMP-2 activity. Gelatin zymography was performed using a 10% polyacrylamide gel containing 0.1% porcine skin gelatin under denaturing conditions (SDS-PAGE), followed by protein renaturation and enzyme activity development. Electrophoresis was carried out at 120 V for 2 h.

The gels were washed twice consecutively for 15 min in washing buffer containing 50 mM Tris-HCl (pH 7.5), 5 mM CaCl_2_, 1 mM ZnCl_2_, and 2.5% (*v*/*v*) Triton X-100 to remove SDS, followed by a brief rinse in the same buffer without Triton X-100. The gels were then incubated at 37 °C for 24 h in developing buffer (50 mM Tris-HCl, pH 7.5; 5 mM CaCl_2_; 1 mM ZnCl_2_; 1% Triton X-100; and 150 mM NaCl). This protocol was adapted from Qi et al. [46]. After incubation, the gels were stained with 0.1% Coomassie Brilliant Blue R-250 at room temperature for 30 min. Finally, enzyme activity was visualized as negative staining using a destaining solution until clear bands appeared. Densitometric quantification of the MMP-2 bands was performed using FIJI software (version 2.9, an open-source distribution of ImageJ (version 1.54p), NIH, Bethesda, MD, USA) [47]. The intensity of the bands was measured by selecting the area corresponding to the active form of MMP-2.

### 2.16. Cell Invasion Assay

Cell invasion assays were performed using 24-well Transwell plates with an 8.0 μm pore size (Corning Inc., Corning, NY, USA). The Transwell chambers were pre-coated with 90 μg/well of Matrigel (Corning Inc., Corning, NY, USA). Briefly, MDA-MB-231 cells (1 × 10^5^ cells/well) in 100 μL of serum-free DMEM were placed in the upper chambers, and 600 μL of DMEM medium containing 5% FBS were added to the lower chamber as a chemoattractant [48]. Cells seeded in the upper chambers were treated with IC_50_ concentrations of δ-cadinene for 24 h. IC_50_ concentrations of Paclitaxel, Quercetin, and 5-FU were used as controls. A culture medium supplemented with 5% FBS and no treatment was used as a positive control. After 24 h of incubation, the cells were fixed with cold methanol for 20 min. The Matrigel, along with the non-invading cells, was removed using a swab. The cells that invaded the membrane were stained with 1% crystal violet for 30 min at room temperature. Photographs were taken using an inverted Nikon Eclipse TS100 microscope equipped with a Nikon DS-U1 photographic system (Olympus, Tokyo, Japan), and the invading cells were quantified using 10% acetic acid. The absorbance was then measured at 595 nm.

### 2.17. Statistical Analysis

Data are presented as the mean ± SEM. All experiments were performed in triplicate and repeated three times. One and two-way ANOVA, and Tukey’s multiple comparison tests were applied for statistical evaluation. Statistical analyses were conducted using GraphPad Prism 8.0 software (GraphPad Software, Inc., Boston, MA, USA). Results were considered statistically significant when the *p*-value was ≤0.05, represented as *p* < 0.05, * *p* < 0.01, ** *p* < 0.001, and *** *p* < 0.0001.

## 3. Results

### 3.1. Validation of 3D MMP-2 and N-Cadherin Models

**Figure 1:**
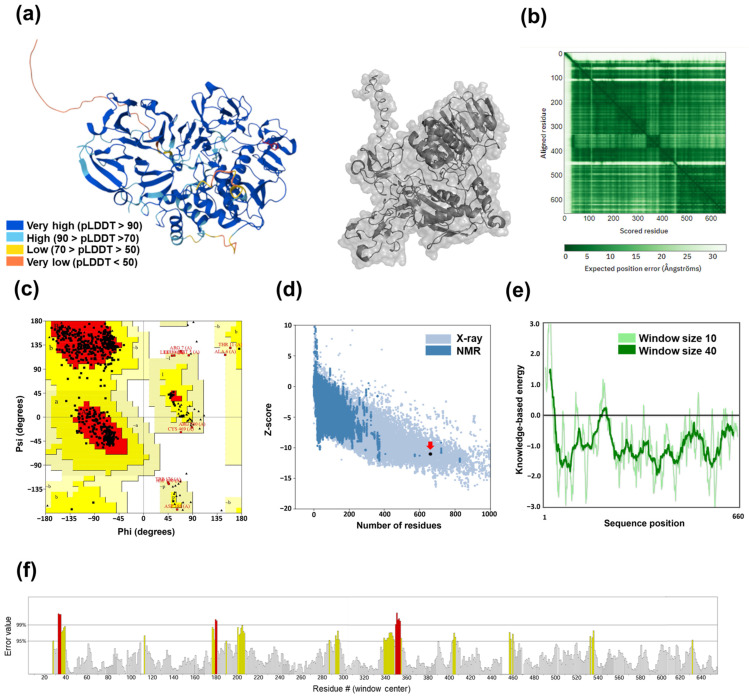
Structural characterization of the MMP-2 protein model. (**a**) Three-dimensional structure of the constructed MMP-2 model. (**b**) Predicted alignment error plot for the AlphaFold MMP-2 model. The X-axis represents residue alignment, and the Y-axis indicates the expected position of residues. (**c**) Ramachandran plot of the MMP-2 3D structure analyzed using the PDBsum platform. Regions of preferred (dark), allowed (light), and disallowed (white) φ/ψ angles are indicated. (**d**) Z-score plot generated using the ProSA-web server. The red arrow indicates the Z-score of the MMP-2 protein. (**e**) Global energy profile based on ProSA-web validation. (**f**) Error quantification using ERRAT. Gray bars represent error-free residues, yellow bars indicate residues with errors in the 95–99% range, and red bars indicate residues with errors greater than 99%.

Given the critical role of invasion and metastasis in BC, we evaluated several key proteins as potential targets for δ-cadinene. CD44, MMP-2, MMP-9, N-cadherin, and ZEB-2 were selected because they complementarily represent the three main axes of early tumor invasion: cell adhesion (CD44 and N-cadherin), extracellular matrix degradation (MMP-2 and MMP-9), and epithelial–mesenchymal transition (N-cadherin) [49,50,51,52]. The 3D models of MMP-2 (Figure 1a,b) and N-cadherin (Supplementary Figure S1) obtained from the AlphaFold Protein Structure Database displayed high stereochemical qualities. The predicted Local Distance Difference Test (pLDDT) scores indicated that most residues exhibited very high (>90) or high confidence (>70), whereas only a minor fraction displayed low or very low confidence (<50), primarily in the flexible or intrinsically disordered regions.

The quality of the MMP-2 and N-cadherin models was evaluated using the PDBsum platform. For the MMP-2 model, 88.3% of the amino acid residues were located in the most favored region, 9.7% in the additionally allowed region, 1.6% in the generously allowed region, and 0.4% in the disallowed region (Figure 1c). For the N-cadherin model, 83.2% of the amino acid residues were in the most favored region, 14.8% in the additionally allowed region, 1.4% in the generously allowed region, and 0.5% in the disallowed region. The Z-scores, calculated using the ProSA-web server, were −10.9 for MMP-2 (Figure 1d,e) and −7.1 for N-cadherin (Supplementary Figure S1), confirming the reliability of the models. Finally, ERRAT validation yielded an overall quality factor of 91.4 for the MMP-2 model and 92.2 for the N-cadherin model (Figure 1f and Supplementary Figure S1). The results obtained from these validation tools demonstrate that the generated models are of excellent quality.

### 3.2. Validation of the Molecular Docking Method

**Figure 2:**
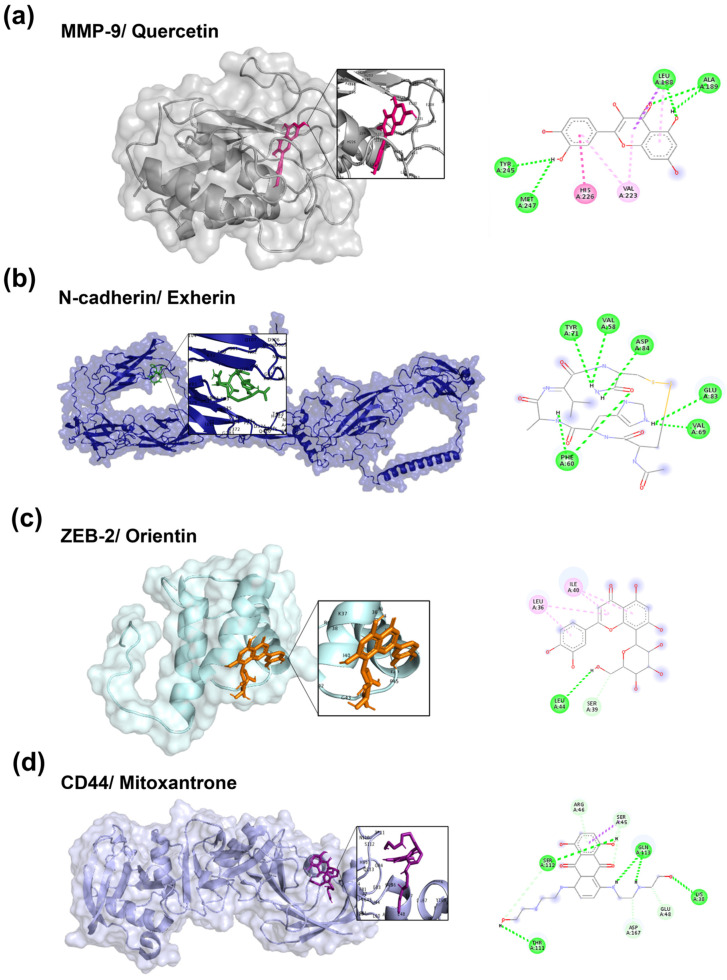
Molecular docking of reference ligands with human proteins implicated in adhesion and migration. (**a**) MMP-9/Quercetin complex, (**b**) N-cadherin/Exherin complex, (**c**) ZEB-2/Orientin complex, and (**d**) CD44/Mitoxantrone complex. For each docking, a close-up view of the ligand binding site is shown together with the 2D diagram of intermolecular interactions: hydrogen bonds (green dotted lines), carbon–hydrogen bonds (cyan dotted lines), π–π stacking interactions (dark pink dotted lines), π–sigma interactions (purple dotted lines), alkyl interactions (pink dotted lines), and π–alkyl interactions (light pink dotted lines).

To validate the docking protocol, a targeted computational study was performed using AutoDock and AutoDock Vina with known reference inhibitors against the active sites of the human cell adhesion and migration proteins. Validation was based on the average docking score, which was calculated as the mean predicted binding energy obtained from both programs. The reference inhibitors showed predicted binding affinities ranging from −4.2 to −8.7 kcal/mol (Figure 2 and Table 1).

Table 1 summarizes the docking results of the tested compounds. Quercetin was evaluated as an inhibitor of MMP-9 and MMP-2. It exhibited the most favorable binding conformation with MMP-9, with an average binding energy of −8.7 kcal/mol. Quercetin interacts with MMP-9 by binding to the active site pocket, forming hydrogen bonds with four residues (Leu188, Ala189, Tyr245, and Met247). Additionally, Quercetin established π–π stacked interactions with His226, π–alkyl interactions with two residues (Leu188 and Val223), and one π–sigma interaction with Leu188. In contrast, Quercetin exhibited an average binding energy of −6.3 kcal/mol with MMP-2, interacting with five hydrophobic residues (Pro105, Ala108, Phe113, Ala194, and Ala196), one amphipathic residue (Tyr182), and one hydrophilic residue (Glu412).

The MD results revealed that Quercetin formed hydrogen bonds with Tyr182, Ala194, and Ala196 (Table 1). Additionally, it established hydrophobic π–anion interactions with Glu412, π–π T-shaped interactions with Phe113, and π–alkyl interactions with Pro105 and Ala108. For N-cadherin, ADH-1 was used as an inhibitor, with an average binding energy of −7.5 kcal/mol. ADH-1 bound within the active site of N-cadherin and interacted with three hydrophobic residues (Val58, Phe60, and Val69) and three hydrophilic residues (Tyr71, Glu83, and Asp84), forming hydrogen bonds. In contrast, Orientin exhibited an average binding energy of −5.2 kcal/mol and bound to the active site pocket of ZEB-2, interacting with three hydrophobic residues (Leu36, Ile40, and Leu44) and one hydrophilic residue (Ser39). Finally, Mitoxantrone was evaluated as an inhibitor of CD44. It showed an average binding energy of −4.2 kcal/mol and was bound within the active site of CD44, forming hydrogen bonds with four hydrophilic residues (Lys38, Thr111, Ser112, and Gln113). Additionally, Mitoxantrone established carbon–hydrogen bonds with four hydrophilic residues (Ser45, Arg46, Glu48, and Asp167), as well as a π–sigma interaction with Ser45.

### 3.3. Molecular Docking Analysis of δ-cadinene

**Figure 3:**
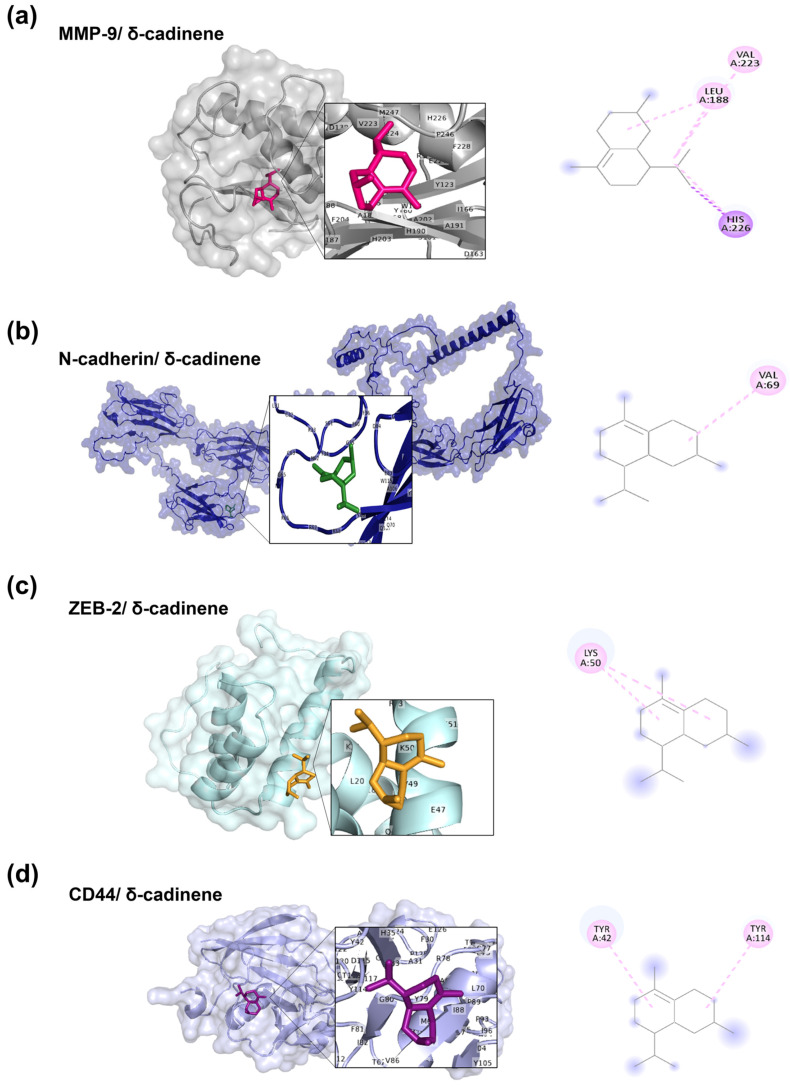
Targeted molecular docking of δ-cadinene with human proteins implicated in adhesion and migration. (**a**) MMP-9/ δ-cadinene complex, (**b**) N-cadherin/ δ-cadinene complex, (**c**) ZEB-2/ δ-cadinene complex, and (**d**) CD44/ δ-cadinene complex. For each docking, a close-up view of the δ-cadinene binding site is shown together with the 2D diagram of intermolecular interactions: π–sigma interactions (purple dotted lines), alkyl interactions (pink dotted lines), and π–alkyl interac-tions (light pink dotted lines).

**Figure 4:**
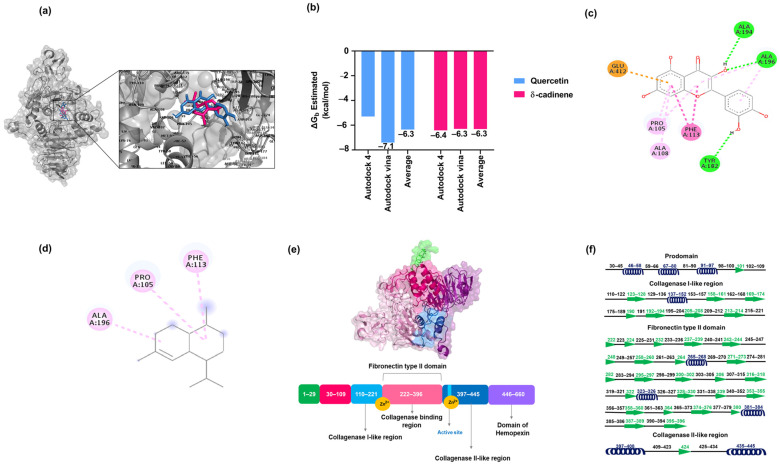
Analysis of interactions of δ-cadinene. (**a**) Binding mode of δ-cadinene and Quercetin at the Quercetin binding site in the human MMP-2 model. (**b**) Comparison of docking scores (ΔG_b_) of δ-cadinene and Quercetin on MMP-2. (**c**) Two-dimensional representation of the interactions obtained by targeted molecular docking of Quercetin: hydrogen bonds (green dotted lines), π–anion interactions (orange dotted lines), π–π stacking interactions (dark pink dotted lines), alkyl interactions (pink dotted lines), and π–alkyl interactions (light pink dotted lines). (**d**) Two-dimensional representation of the interactions obtained by targeted molecular docking of δ-cadinene: alkyl interactions (pink dotted lines) and π–alkyl interactions (light pink dotted lines). (**e**) Representation of the constructed MMP-2 model and sequence. (**f**) Structural alignment of the prodomain, Collagenase I-like domain, Fibronectin type II domain, and Collagenase II-like domains of MMP-2. Black lines represent loops, blue spirals represent α-helices, and green arrows represent β-sheets.

After validation with various reference inhibitors, molecular-targeted docking analysis was performed on δ-cadinene, focusing on the active sites previously characterized for each evaluated protein. The MD analyses (Figure 3) revealed that δ-cadinene was consistently accommodated within the catalytic pockets of MMP-9, N-cadherin, ZEB-2, and CD44. AutoDock and AutoDock Vina predicted stable conformations with binding free energies comparable to or exceeding the cut-off thresholds defined by reference inhibitors. The MD analysis revealed that δ-cadinene interacts with the Quercetin binding site on MMP-2 (Figure 4a), exhibiting an average binding energy of −6.3 kcal/mol (Figure 4b and Table 2), which is equivalent to that of Quercetin with MMP-2 (Table 1). Intermolecular interaction analysis revealed that δ-cadinene primarily engaged with three hydrophobic residues (Pro105, Phe113, and Ala196) through alkyl and π–alkyl interactions (Figure 4c,d).

A detailed structural analysis of MMP-2 revealed the presence of three Fibronectin type II collagen-binding domains, a hemopexin repeat, and a matrixin motif within its catalytic site, all of which are essential for enzymatic activity (Figure 4e,f). Interestingly, δ-cadinene was found to bind specifically to the collagenase-binding region of MMP-2, which is a fundamental site for its protease function. Moreover, δ-cadinene also interacted with the Quercetin binding site on MMP-9, exhibiting an average binding energy of −6.2 kcal/mol, where it established alkyl interactions with two hydrophobic residues (Leu188 and Val223), as well as π–alkyl and π–sigma interactions with the amphipathic residue His226.

Otherwise, δ-cadinene interacted with the ADH-1 binding site on the N-cadherin model, exhibiting an average binding energy of −5.9 kcal/mol and forming alkyl interactions with one hydrophobic residue (Val69). Conversely, δ-cadinene bound to the Orientin site on ZEB-2, with an average binding energy of −4.6 kcal/mol, establishing alkyl interactions with one hydrophobic residue (Lys50). Finally, at the Mitoxantrone binding site on CD44, δ-cadinene showed an average binding energy of −4.5 kcal/mol and formed hydrophobic π–alkyl interactions with two aromatic residues (Tyr42 and Tyr114).

Collectively, these results suggest that δ-cadinene has a higher binding affinity for MMP-2 (−6.3 kcal/mol) than for MMP-9 (−6.2 kcal/mol), which correlates with the greater number of intermolecular interactions detected.

### 3.4. Blind Molecular Docking Analysis of δ-cadinene

**Figure 5:**
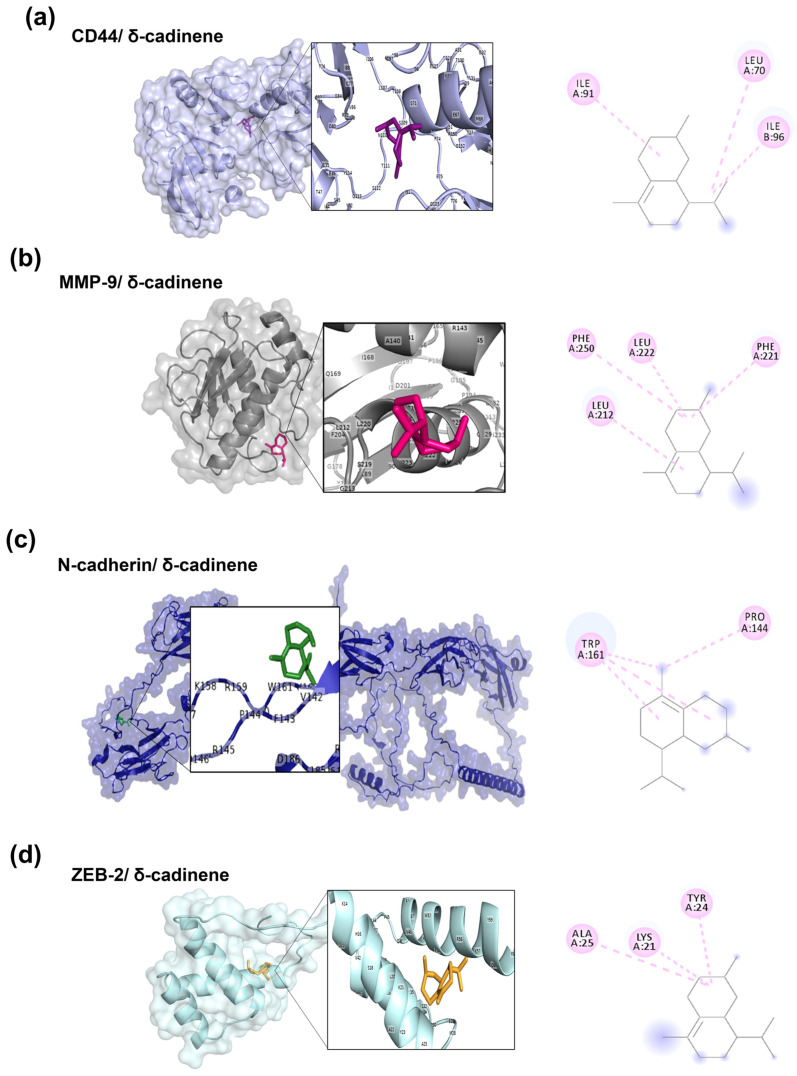
Blind docking studies of δ-cadinene with human proteins involved in adhesion and mi-gration. (**a**) CD44/ δ-cadinene complex, (**b**) MMP-9/ δ-cadinene complex (**c**) N-cadherin/δ-cadinene complex, and (**d**) ZEB-2/δ-cadinene complex. For each complex, the binding site is highlighted with a close-up view of the ligand within the protein, together with the corresponding 2D interaction diagram. Interaction types are shown in color: π–sigma (purple dotted lines), alkyl (pink dotted lines), and π–alkyl (light pink dotted lines).

As the binding sites of δ-cadinene on these proteins were unknown, AutoDock Vina was employed to perform blind docking studies as a complementary approach. The resulting docking poses, interaction patterns, and binding affinities of δ-cadinene with the target proteins are illustrated in Figure 5 and summarized in Table 3, respectively. Based on the blind docking results, δ-cadinene bound to different pockets of human cell adhesion and migration proteins. For CD44, a protein that facilitates cell adhesion and pro-invasive signaling [50], δ-cadinene exhibited a binding energy of −6.4 kcal/mol and engaged with three hydrophobic residues (Leu70, Ile91, and Ile96) through alkyl interactions. Similarly, δ-cadinene showed a binding energy of −6.0 kcal/mol with MMP-9, forming alkyl and π–alkyl interactions with four hydrophobic residues (Leu212, Phe221, Leu222, and Phe250).

In the case of N-cadherin, which mediates interactions between cancer cells and cancer-associated fibroblasts, thereby promoting tumor invasion and progression [51], δ-cadinene exhibited a binding energy of −6.0 kcal/mol and interacted with two hydrophobic residues (Pro144 and Trp161) via alkyl and π–alkyl interactions, respectively. Conversely, δ-cadinene bound to ZEB-2 with a binding energy of −5.6 kcal/mol, engaging with two hydrophobic residues (Lys21 and Ala25) and one amphipathic residue (Tyr24) through alkyl and π–alkyl interactions.

**Figure 6:**
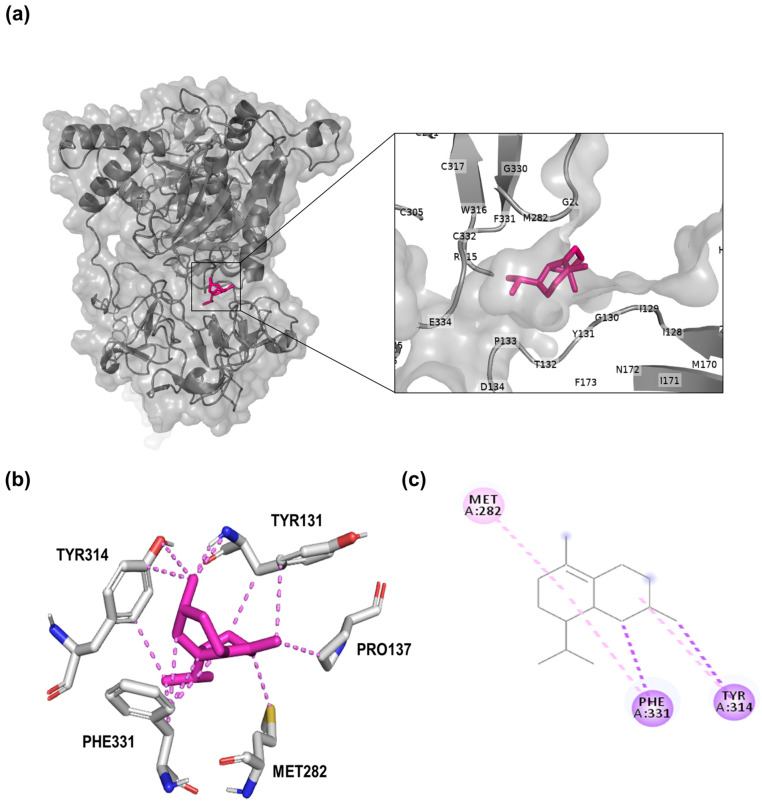
Blind molecular docking analysis between δ-cadinene and MMP-2 protein. (**a**) Representation of the X-ray crystal structure of human MMP-2 (gray cartoon) bound to δ-cadinene (pink sticks), with a magnified view of the binding site showing the relevant amino acids in greater detail. (**b**) Five amino acid residues of MMP-2 interact with δ-cadinene: three hydrophobic (Pro137, Met282, and Phe331) and two amphipathic (Tyr131 and Tyr314). (**c**) Two-dimensional representation of the interactions identified between MMP-2 and δ-cadinene, showing alkyl interactions (pink dotted lines), π–alkyl interactions (light pink dotted lines), and two π–sigma interactions (purple dotted lines).

This protein activates NF-κB and PI3K/Akt signaling pathways, which are essential for tumor progression [50]. The results showed that the most favorable binding site for δ-cadinene was identified in MMP-2. It was confirmed that δ-cadinene binds to the previously reported active site of the Collagenase region II of the MMP-2 protein, with a binding energy of −7.7 kcal/mol. Nevertheless, δ-cadinene binds to a novel pocket on MMP-2 (Figure 6a and Table 3), corresponding to the Fibronectin type II domain, suggesting potential inhibition of MMP-2 activity. Figure 6b shows the predicted interaction profile of δ-cadinene, generated using the PyMOL program, which revealed interactions with three hydrophobic residues (Pro137, Met282, and Phe331) and two amphipathic residues (Tyr131 and Tyr314).

Conversely, δ-cadinene bound to ZEB-2 with a binding energy of −5.6 kcal/mol, engaging with two hydrophobic residues (Lys21 and Ala25) and one amphipathic residue (Tyr24) through alkyl and π–alkyl interactions. This protein activates NF-κB and PI3K/Akt signaling pathways, which are essential for tumor progression [52]. The results showed that the most favorable binding site for δ-cadinene was identified in MMP-2.

It was confirmed that δ-cadinene binds to the previously reported active site of the Collagenase region II of the MMP-2 protein, with a binding energy of −7.7 kcal/mol. Nevertheless, δ-cadinene binds to a novel pocket on MMP-2 (Figure 6a and Table 3), corresponding to the Fibronectin type II domain, suggesting potential inhibition of MMP-2 activity. Figure 6b shows the predicted interaction profile of δ-cadinene, generated using the PyMOL program, which revealed interactions with three hydrophobic residues (Pro137, Met282, and Phe331) and two amphipathic residues (Tyr131 and Tyr314). Further analyses of the δ-cadinene–MMP-2 interactions were conducted using Discovery Studio 2024 to provide additional details. This analysis indicated that δ-cadinene formed alkyl interactions with one hydrophobic residue (Met282) and π-sigma interactions with another hydrophobic residue (Phe331). Additionally, it established π-sigma and π-alkyl interactions with one amphipathic residue (Tyr314) (Figure 6c and Table 3). These findings demonstrate the high affinity of δ-cadinene for cell adhesion and migration proteins, supporting its potential role in inhibiting or altering the mechanisms of invasion and migration.

To evaluate whether the presence of Zn^2+^ could modify the structure and binding site of δ-cadinene, blind docking was performed on the MMP-2–Zn^2+^ model complex. Independently, the interactions were observed at the same binding site, which was confirmed by the superposition of the complexes, showing that δ-cadinene was consistently bound to the same site in both cases. The binding energies were comparable (≈−7.7 kcal/mol), equivalent to those obtained with the Zn^2+^-free model. These findings indicate that the presence of Zn^2+^ does not affect either the three-dimensional conformation of the protein or the affinity of δ-cadinene for its preferential binding site (Supplementary Figure S2). Therefore, we used the Zn^2+^-free model as the reference for subsequent analyses, as it provides an equally valid structural representation while being methodologically simpler and consistent with previously reported models [53].

### 3.5. Molecular Dynamics Analysis of Simulations

**Figure 7:**
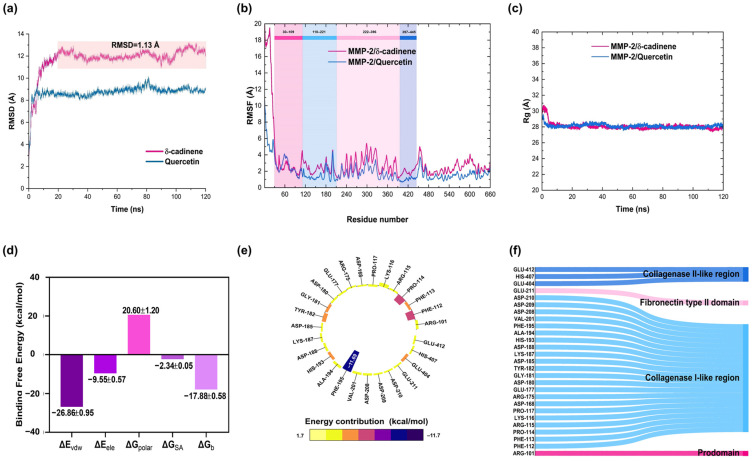
Molecular dynamic simulations of the complex MMP-2 (Collagenase binding region)/δ-cadinene. (**a**) RMSD values of the MMP-2/δ-cadinene and MMP-2/Quercetin complex. (**b**) RMFD analysis showing domain-specific flexibility within each complex. The colors indicate the following: dark pink highlights fluctuations within the prodomain, light blue represents fluctuations in the Collagenase I-like region, light pink corresponds to fluctuations in the Fibronectin type II domain, and dark blue indicates fluctuations within the Collagenase II-like region. (**c**) Rg analysis of both complexes over the simulation time. (**d**) Binding free energy (kcal/mol) of the MMP-2/δ-cadinene targeted complex, calculated using MMPBSA method. The bar plots show energy components contribution: van der Waals contributions (∆E_vdw_), electrostatic contributions (∆E_ele_), polar contributions (∆G_polar_), SASA contributions (∆G_SA_), and affinity energy (∆G_b_). (**e**) Per-residues free energy decomposition of the MMP-2/δ-cadinene targeted complex. A heat map displays the contribution of each residue to ligand binding, where yellow indicates the highest values and purple indicating the lowest. Only residues with a ΔG_b_ < −0.5 kcal/mol and ΔG_b_ > 0.5 kcal/mol are shown. (**f**) Spatial localization of the key amino acid residues contributing to ligand binding within the MMP-2 protein.

The complexes with the most favorable energies were selected for MDS based on the results of both targeted and blind molecular docking studies of the cell adhesion and migration proteins. GROMACS version 2018.4 with the AMBER03 force field was used to assess the conformational stability of the complexes over a defined simulation period. Using the atomic coordinates obtained from the docking simulations, a total simulation time of 120 ns was achieved. Initially, we evaluated the stability of the complexes formed between δ-cadinene or Quercetin and human MMP-2. The RMSD analysis of the MMP-2/Quercetin complex (blue) showed stabilization beginning at 20 ns, with minimum and maximum oscillations of 0.0055 Å and 10.17 Å, respectively, resulting in a total fluctuation of 10.16 Å over the 120 ns simulation (Figure 7a). In contrast, the MMP-2/δ-cadinene complex was stabilized within the first 20 ns, exhibiting a mean fluctuation of 1.13 Å. After 120 ns, the oscillation difference in the MMP-2/δ-cadinene complex (pink) reached 13.22 Å, with a minimum oscillation of 0.0053 Å and a maximum of 13.23 Å (Figure 7a), demonstrating the proper equilibration of the complex during the simulation. Additionally, RMSF analysis was performed to identify the local movements of individual residues throughout the 120 ns of MDS. The MMP-2/δ-cadinene complex exhibited a maximum fluctuation of 20.31 Å and an average fluctuation of 3.07 Å (Figure 7b), whereas the MMP-2/Quercetin complex displayed a maximum fluctuation of 10.73 Å and an average fluctuation of 1.85 Å.

In addition, the Rg was evaluated for the δ-cadinene and Quercetin complexes to assess the folding and compactness of the protein–ligand systems during the simulation (Figure 7c). The graph comparing the MMP-2/δ-cadinene and MMP-2/Quercetin complexes shows that both maintained most of their initial compactness throughout the simulation, exhibiting minimal fluctuations and mean Rg values of 28.11 and 28.06 Å, respectively. These results support the reliability of the molecular docking predictions, as they demonstrate the stability of the interactions between δ-cadinene or Quercetin with MMP-2. Additionally, MMPBSA calculations were performed to estimate the ΔG_b_ of the protein/ligand complexes during MDS, providing valuable information on molecular recognition. The δ-cadinene complex had a ΔG_b_ value of −18.0 ± 0.6 kcal/mol (Figure 7d). Furthermore, the residue decomposition of the binding energy contributions was performed over the last 10 ns of the MDS using g_mmpbsa to identify the key residues involved in the MMP-2/δ-cadinene interaction.

**Figure 8:**
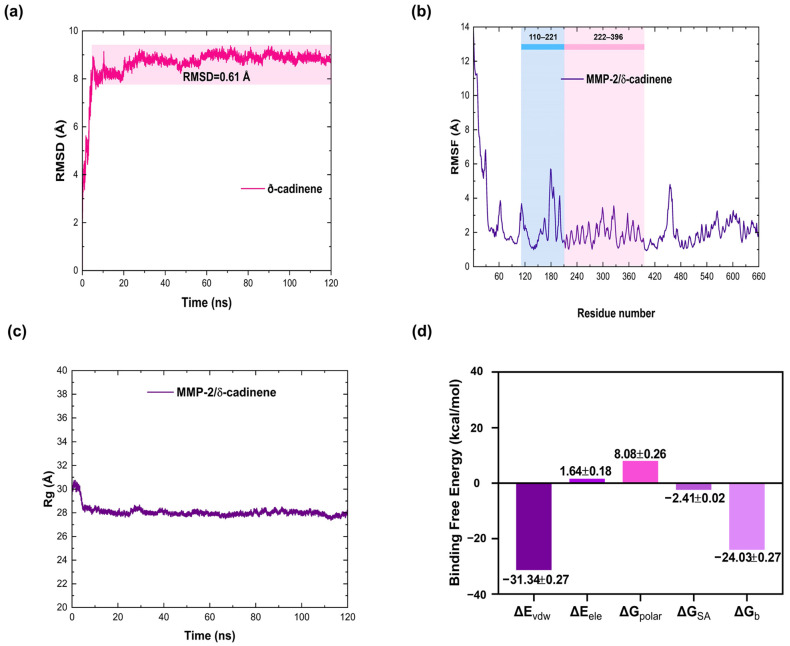
Molecular dynamics simulation of the blind MMP-2/δ-cadinene complex in the Fibronectin type II domain. (**a**) RMSD profile, with the pink box highlighting the average oscillations between 6 and 120 ns. (**b**) RMSF values for protein residues, where light blue indicates fluctuations in the Collagenase I-like region and light pink highlights fluctuations in the Fibronectin type II domain. (**c**) Rg analysis of the MMP-2/δ-cadinene complex. (**d**) Binding free energy (kcal/mol) of the blind MMP-2/δ-cadinene complex, calculated using the MM-PBSA method. Bar plots show the contribution of energy components: van der Waals (∆_Evdw_), electrostatic (∆_Eele_), polar (∆G_polar_), SASA (∆_GSA_), and binding free energy (∆G_b_).

The results showed that 27 amino acid residues, primarily located in the Collagen-binding region of MMP-2, interacted with δ-cadinene, with residue Phe195 contributing most favorably to the ΔG_b_ value (−11.69 kcal/mol; Figure 7e,f). As mentioned previously, blind molecular docking results for human MMP-2 revealed that δ-cadinene binds to a different site on the protein. As shown in Figure 8a, the stability of the MMP-2/δ-cadinene blind complex was analyzed. δ-cadinene exhibited minimum and maximum oscillations of 0.0054 Å and 9.37 Å, respectively. The complex stabilized within the first 6 ns, with a mean fluctuation of 0.61 Å. Regarding RMSF, the δ-cadinene/MMP-2 blind complex showed a maximum fluctuation of 13.74 Å and an average fluctuation of 2.33 Å (Figure 8b). The Rg analysis (Figure 8c) revealed a mean radius of gyration of 28.06 Å, indicating the stable compactness of the complex. Consistently, the MMPBSA analysis of the MMP-2/δ-cadinene blind complex estimated a ΔG_b_ value of −24.0 ± 0.3 kcal/mol (Figure 8d). These results confirm the formation of stable interactions between δ-cadinene and MMP-2, thereby strengthening the reliability of the molecular docking predictions.

**Figure 9:**
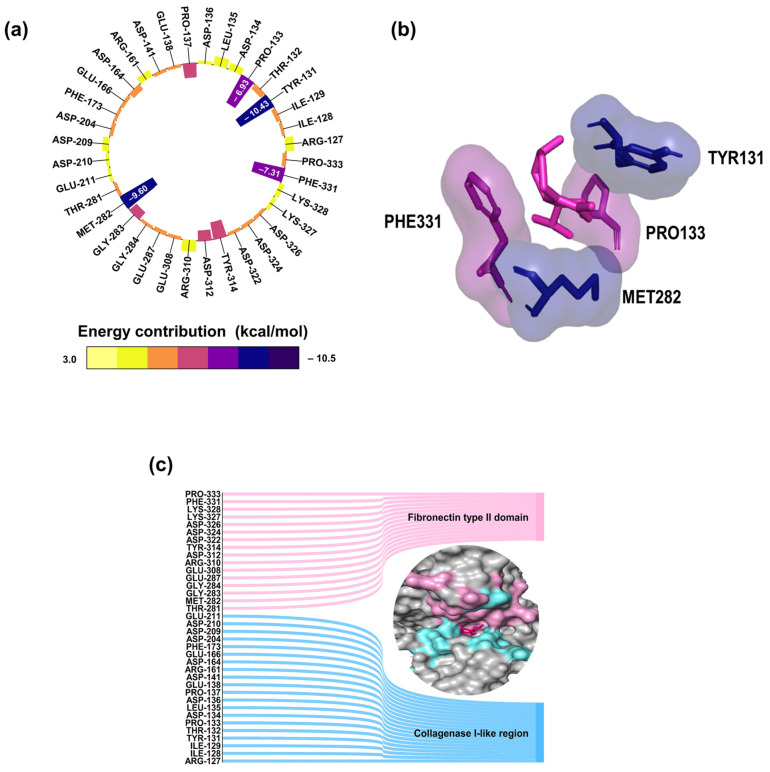
Molecular dynamic simulations of the complex MMP-2 (Fibronectin type II domain/δ-cadinene. (**a**) Heatmap of per-residue free energy decomposition for the MMP-2/δ-cadinene blind complex. Highlighting amino acid residues contributing most to ligand binding, Yellow represents the highest contributions, while purple indicates the lowest. Only residues with ΔG_b_ < −0.5 kcal/mol and ΔG_b_ > 0.5 kcal/mol were included. (**b**) Spatial representation of key amino acid residues contributing to the binding energy of δ-cadinene at the MMP-2 interaction interface. (**c**) Binding site prediction from blind docking. MMP-2’s surface is shown in gray, with the Fibronectin type II domain highlighted in light pink, the Collagenase I-like region in light blue, and the δ-cadinene represented in pink.

As shown in Figure 9a,b, residue decomposition analysis revealed that four residues contributed most favorably to the ΔG_b_ value: Pro133 (−7.0 kcal/mol) and Tyr131 (−10.4 kcal/mol), located in the Collagenase I-like region, and Phe331 (−7.3 kcal/mol) and Met282 (−9.6 kcal/mol), located in the Fibronectin type II domain. Furthermore, 36 amino acid residues within these two regions of MMP-2 were identified to interact with δ-cadinene (Figure 9c).

### 3.6. Effect of δ-cadinene on Cell Viability of MDA-MB-231 and MCF10-A Cell Lines

**Figure 10:**
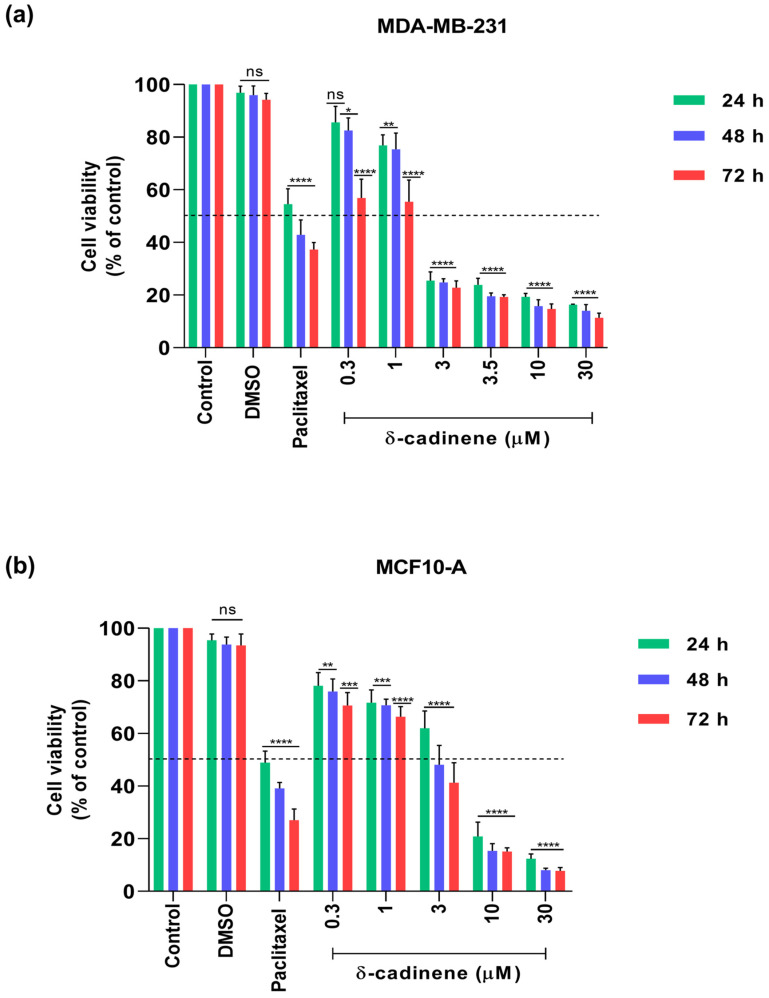
Effect of δ-cadinene on the viability of MDA-MB-231 and MCF10-A cells. (**a**) MDA-MB-231 and (**b**) MCF10-A cells were treated with 0.2% DMSO, 0.3 µM paclitaxel, and increasing concentrations of δ-cadinene (0.3–30 µM) for 24, 48, and 72 h. Cell viability was measured by MTT assay. Results are expressed as mean ± SEM of n = 3 per group. Two-way analysis of variance (ANOVA), followed by Tukey’s multiple comparisons test, was performed. Statistical significance: * *p* < 0.05, ** *p* < 0.01, *** *p* < 0.001, **** *p* < 0.0001; ns = not significant compared to control.

We evaluated the cytotoxic effects of δ-cadinene (0.3–30 μM) on MDA-MB-231 and MCF10-A cells. As shown in Figure 10, δ-cadinene progressively reduced the viability of MDA-MB-231 cells in a time-dependent manner (after 24, 48, and 72 h).

After 24 h of treatment, the IC_50_ value for MDA-MB-231 cells was 1.7 ± 0.1 μM, after 48 h, it remained at 1.7 ± 0.1 μM; and after 72 h, it further decreased to 0.6 ± 0.1 μM. Paclitaxel exhibited a significantly greater cytotoxic effect than δ-cadinene at the same concentration (0.3 μM) (Figure 10a). Paclitaxel also reduced the viability of MCF10-A breast epithelial cells line (Figure 10b), highlighting its harmful effects on normal cells. In contrast, δ-cadinene displayed higher IC_50_ values on MCF10-A cells: 3.2 ± 0.1 μM at 24 h, 2.2 ± 0.1 μM at 48 h, and 1.6 ± 0.1 μM at 72 h. A marked decrease in viability was observed between 1 and 3 μM in the MDA-MB-231 cell line.

These results demonstrate that δ-cadinene significantly reduced cell viability in a concentration- and time-dependent manner. Additionally, the PaccMann platform predicted a theoretical IC_50_ value of 3.5 μM for δ-cadinene in MDA-MB-231 cells. To validate this prediction, we performed MTT assays a fixed concentration of 3.5 μM (Figure 10a). However, experimental results showed that treatment with 3.5 μM δ-cadinene reduced cell viability by 76.2% at 24 h, 80.5% at 48 h, and 80.7% at 72 h, which were substantially lower than the expected 50% threshold. These findings suggest that the model-predicted concentration may underestimate the actual cytotoxic potential of δ-cadinene in vitro (Figure 10a). According to the SI criteria, δ-cadinene exhibited an SI > 1 against MDA-MB-231 cells at 24, 48, and 72 h (Table 4), indicating selective cytotoxicity toward BC cells compared to non-tumorigenic MCF10-A cells.

### 3.7. Effect of δ-cadinene on the Morphology of MDA-MB-231 and MCF10-A Cell Lines

To assess whether the reduction in cell viability observed in MDA-MB-231 and MCF10-A cells was accompanied by morphological alterations, control cells were stained with H & E and examined under light microscopy. The analysis revealed that the control cells exhibited normal morphology and grew as a confluent monolayer.

**Figure 11:**
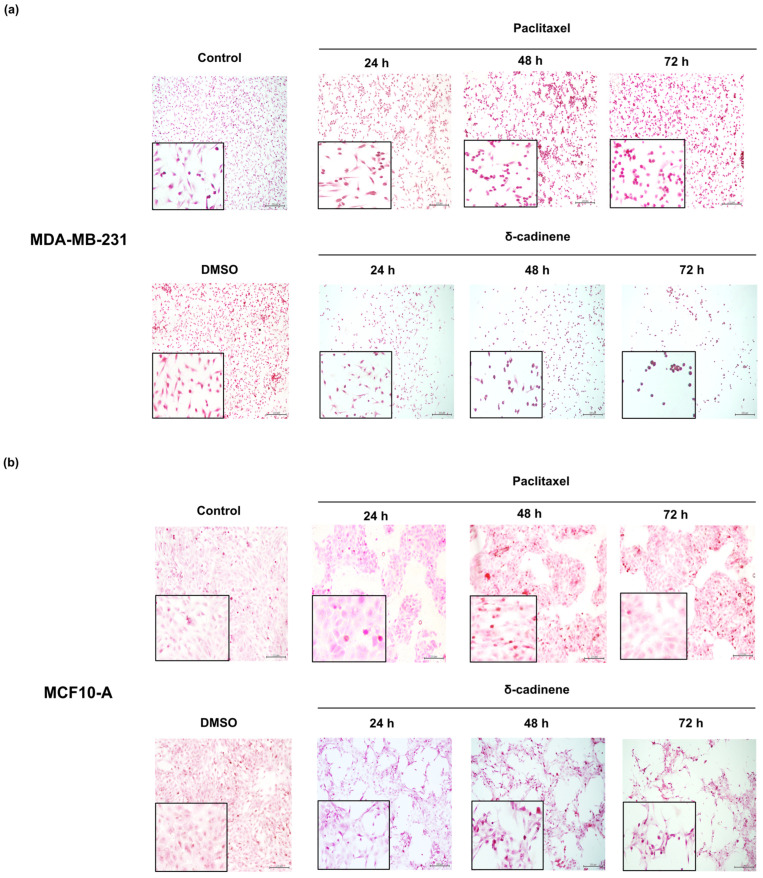
δ-cadinene promotes morphological changes in MDA-MB-231 BC cells. Representative images of H & E staining. (**a**) MDA-MB-231 and (**b**) MCF10-A cells treated with their respective IC_50_ concentrations of δ-cadinene and paclitaxel for 24, 48, and 72 h. Images were acquired at 10× magnification. Insets show magnified views of the regions indicated by the black boxes. Scale bar = 200 μm.

The morphological analysis was conducted to evaluate the structural changes in MDA-MB-231 and MCF10-A cells following treatment. Cells exposed to the vehicle control (0.2% DMSO) maintained normal morphology in both cell lines, with no evidence of cytoplasmic retraction, loss of polarity, or cell rounding (Figure 11a,b). In contrast, Paclitaxel treatment induced pronounced morphological alterations: MDA-MB-231 cells (Figure 11a) displayed a rounded morphology consistent with cytoskeletal disruption, whereas MCF10-A cells exhibited cytoplasmic retraction and loss of their characteristic epithelial polarity. Untreated MDA-MB-231 cells (Figure 11a) retained their typical growth pattern, consisting of pleomorphic cells with irregularly shaped nuclei, spindle-shaped morphology, and an elongated appearance.

Conversely, MDA-MB-231 cells treated with the IC_50_ concentration of δ-cadinene showed reduced confluence and marked morphological alterations after 24 h of incubation, including decreased cell size, membrane blebbing, and shrinkage. These changes became more pronounced over time, and by 48 and 72 h, the cells exhibited a fully rounded morphology. Untreated MCF10-A cells (Figure 11b), maintained an organized monolayer with well-defined edges, despite exhibiting natural variations in nuclear and cellular size and shape. However, after 24 h of exposure to δ-cadinene at the IC_50_, MCF10-A cells progressively lost intercellular contact and formed disorganized structures. By 48 and 72 h, the cells exhibited contraction of their epithelial morphology, adopting an abnormal rounded shape accompanied by evidence of plasma membrane disruption.

### 3.8. Effect of δ-Cadinene on the Invasion of the MDA-MB-231 Cell Line

To further investigate whether δ-cadinene suppress the invasive potential of BC cells, a Transwell invasion assay was performed using the MDA-MB-231 cell line. For comparison, cells were treated with the IC_50_ concentrations of Paclitaxel, Quercetin, and 5-FU, while untreated cultures maintained in medium supplemented with 5% FBS served as a positive control.

**Figure 12:**
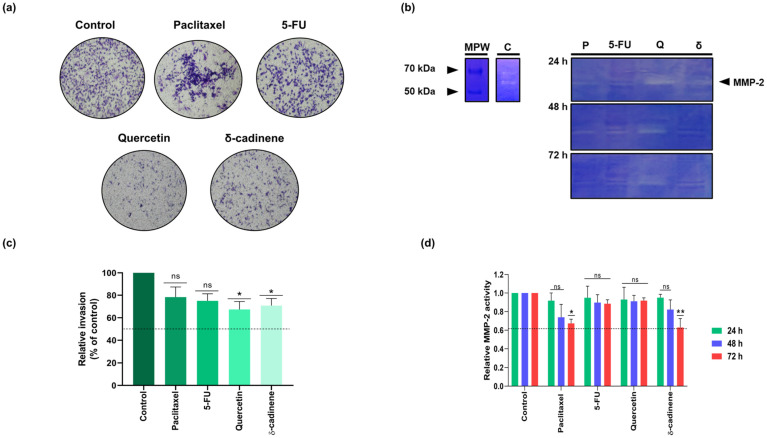
δ-cadinene decreases the invasion and MMP-2 activity in the MDA-MB-231 BC cells. (**a**) Representative images of Transwell invasion assays in MDA-MB-231 cell line treated with the IC_50_ concentrations of compound δ-cadinene, Paclitaxel, 5-FU, and Quercetin for 24 h. Images were captured at 10× magnification using a Nikon Eclipse TS100 inverted microscope equipped with a Nikon DS-U1 digital imaging system. (**b**) MMP-2 protease activity. MDA-MB-231 cells were treated with IC_50_ concentrations of δ-cadinene for 24, 48 and 72 h. The representative zymography blots show the activity of MMP-2. Abbreviations: MWM, molecular weight marker; C, control; P, Paclitaxel; 5-FU, 5-Fluorouracil; Q, Quercetin; δ, δ-cadinene. (**c**) Quantification of relative cell invasion was performed by measuring the absorbance of solubilized crystal violet retained by invaded cells. Data are presented as mean ± SEM (n = 3 per group). Statistical analysis was performed using One-way analysis of variance (ANOVA) followed by Tukey’s test. *p* < 0.05 (*); ns = not significant, compared to control. (**d**) Quantification of relative MMP-2 activity. Data are presented as mean ± SEM (n = 3 per group). Statistical analysis was performed using Two-way analysis ANOVA followed by Tukey’s test. *p* < 0.05 (*), *p* < 0.01 (**); ns = not significant, compared to control. The data were normalized to the protein content on the plates and are presented in respective graphs.

As shown in Figure 12a,c, δ-cadinene and Quercetin significantly reduced the invasion of MDA-MB-231 cells after 24 h of incubation compared to the untreated control (*p* < 0.05). In contrast, Paclitaxel (*p* = 0.1378) and 5-FU (*p* = 0.0578) did not induce statistically significant changes at this time point.

Additionally, gelatin zymography was performed to assess the effect of the treatments on MMP-2 enzymatic activity in MDA-MB-231 cells at 24, 48, and 72 h. Gelatinolytic bands corresponding to the active form of MMP-2 were visualized and quantified by densitometric analysis using the FIJI software. Band intensities were normalized to the untreated control and expressed as relative enzymatic activity. As shown in Figure 12b,c, Paclitaxel treatment resulted in a reduction in MMP-2 activity across all time points; however, statistical significance was achieved only at 72 h (*p* < 0.05). Similarly, 5-FU treatment led to a modest decrease in MMP-2 activity at later time points, although these changes were not significant. Quercetin did not alter the relative MMP-2 gelatinolytic activity compared to that in the untreated group.

Notably, δ-cadinene induced a progressive, time-dependent reduction in MMP-2 activity, with significant inhibition observed at 72 h (*p* < 0.01), suggesting a delayed but biologically meaningful impact on the invasive machinery of BC cells (Figure 12d). Collectively, these results indicate that, among the compounds tested, only Paclitaxel and δ-cadinene achieved a significant reduction in MMP-2 activity after prolonged exposure, reinforcing the potential of δ-cadinene as a selective anti-invasive agent.

## 4. Discussion

BC is currently the most common malignant tumor in women worldwide. Treatments are often invasive and non-specific, leading to significant side effects for patients [54]. EOs are volatile mixtures of plant secondary metabolites produced in leaves, bark, seeds, and fruits, and are typically distinguished by their characteristic scent [13]. Numerous studies have demonstrated their broad spectrum of biological activities, including antimicrobial, antioxidant, analgesic, repellent, anti-inflammatory, and anticancer properties [14]. Despite this potential, their clinical application faces major challenges due to the high volatility and poor aqueous solubility of their metabolites, the large variability in the composition of EOs, and the presence of several compounds that may cause adverse effects [15]. These limitations emphasize the need to identify and characterize the specific metabolites that can cause adverse effects, as well as to explore advanced delivery strategies, such as nanoemulsions or nanoparticle-based systems, to improve their stability and bioavailability [55]. Currently, several studies have shown that secondary metabolites have significant potential for the treatment of BC [56]. Several studies have demonstrated that terpenoids possess significant therapeutic potential in both the prevention and treatment of BC [57].

These secondary metabolites, known for their structural diversity and bioactivity, have been shown to modulate key signaling pathways involved in tumor growth, apoptosis, and metastasis [56,57]. δ-cadinene is a terpene, specifically from the group of bicyclic sesquiterpenes, found in the EOs of many plants, such as *D. bicolor* [19]. It shows significant cytotoxic activity against cancer cells in vitro. Hui et al. [21] found that δ-cadinene induced both concentration-and-time-dependent growth-inhibitory effects on the OVCAR-3 cell line. Additionally, δ-cadinene caused cell cycle arrest in the sub-G1 phase and induced features characteristic of apoptosis, including cell shrinkage, chromatin condensation, and nuclear membrane rupture. Treatment with 50 and 100 µM of δ-cadinene resulted in a significant increase in caspase-9 expression levels. However, the mechanism of δ-cadinene in BC remains unknown. This study aimed to investigate the efficacy of δ-cadinene in inhibiting BC cell invasion both in silico and in vitro. For the first time, we report the cytotoxic effect of the metabolite δ-cadinene in triple-negative BC, identifying MMP-2 as one of its potential molecular targets that contributes to a significant reduction in cancer cell invasion. In our study, MDS were performed to evaluate the ability of δ-cadinene to interact with the active sites of proteins involved in cell adhesion and invasion.

Accurate prediction of protein structure is essential for understanding molecular interactions and developing effective therapeutic strategies. In this study, the 3D models of MMP-2 (UniProt ID: P08253) and N-cadherin (UniProt ID: P19022) were retrieved from the AlphaFold Protein Structure Database. These predicted structures, which contained regions suitable for structural analyses with high confidence, provided reliable templates for subsequent docking and MDS, supporting the investigation of interactions relevant to cell adhesion and migration in BC. To ensure the reliability of the models, we performed a thorough validation using established tools such as PDBsum, ProSA-web, and ERRAT.

The PDBsum analysis revealed that 88.3% of the residues in the MMP-2 model fell in the most favored regions of the Ramachandran plot, while 83.2% of the residues in the N-cadherin model fell within these regions. These results indicate that the models have an appropriate stereoelectronic conformation [26,58,59,60,61]. In addition, the Z-scores of MMP-2 determined with ProSA-web were −10.92 and of N-cadherin, −7.09. These values are within the expected range for native proteins of similar size, suggesting adequate structural stability. ERRAT validation yielded overall quality factors of 91.4% for MMP-2 and 92.2% for N-cadherin, exceeding the 90% threshold normally associated with high-quality structural models. Recent studies have modelled the structures of MMP-2 and N-cadherin using the AlphaFold Protein Structure Database. These validation metrics agree with those of other studies, suggesting that our models are of comparable quality [58,59,62,63]. Our validation results confirm the high structural quality of the models, and support their suitability for subsequent MD and MDS. Accurate prediction of MMP-2 and N-cadherin structures is essential for understanding their interactions with inhibitory drugs and for the development of targeted therapeutic strategies. Therefore, computational modelling was used when experimental structures were not available. For example, in the case of Quercetin binding to MMP-9, three key interacting residues (Leu188, Ala189, and Met247) matched those reported by Huynh et al. [36]. However, when analyzing the binding Quercetin to MMP-2, differences were found that are likely due to the fact that were used a full-length protein model generated with the AlphaFold Protein Structure Database, as opposed to the experimental partial structures used in previous studies [36]. Also, N-cadherin model, showed different residues interacting residues with ADH-1 compared to previous reports [33], suggesting possible alternative binding conformations. Orientin also showed different binding patterns when it docked to ZEB-2 [35], and in the case of CD44, Mitoxantrone was found to bind to Gln133, a residue previously identified as critical by other authors [34].

These results demonstrate the importance of using comprehensive, high-quality structural models to accurately define potential binding interfaces and increase the reliability of docking. After validation, targeted docking studies were performed to evaluate the binding affinity of δ-cadinene with the active sites of proteins involved in cell adhesion and invasion. Our MD results consistently identified MMP-2 as a high-affinity target for δ-cadinene. In targeted docking studies, δ-cadinene exhibited an average binding energy comparable to that of Quercetin and interacted with key residues of the catalytic site, such as Pro105, Phe113, and Ala196 [37,64]. These common interactions suggest that δ-cadinene binds analogously to Quercetin and may have a similar inhibitory profile. However, blind docking studies showed a shift in the favored binding site towards the Fibronectin type II domain of MMP-2, where δ-cadinene had an even lower binding energy. The Fibronectin type II domain is known to mediate interactions with EMC components, particularly Collagen IV, and plays a crucial role in substrate recognition [65]. The localization of δ-cadinene to this domain suggests a potential allosteric mechanism of inhibition, possibly affecting the adhesion and matrix-binding capabilities of MMP-2 rather than directly blocking its catalytic activity. These results extend the understanding of the inhibitory profile of δ-cadinene and emphasize the importance of considering non-catalytic domains as relevant therapeutic targets in metastasis-related processes.

As shown in Figure 7a, the Rg values of the MMP-2/δ-cadinene and MMP-2/Quercetin complexes remained stable over the 120 ns MDS, confirming the structural integrity of both the targeted and blind complexes. However, their binding profiles differed: in the targeted docking approach (Figure 4a), δ-cadinene bound to the predefined active site, whereas in the blind docking analysis (Figure 3a), it was located in alternative pockets, suggesting possible allosteric interactions.

The RMSF analysis of the MMP/δ-cadinene and MMP-2/Quercetin complexes (Figure 7b) showed minimal fluctuation in most regions, with the exception of one segment corresponding to a flexible loop, identified by structural alignment (Figure 4f). The observed similarity of the fluctuation patterns between δ-cadinene and Quercetin with only minor differences, indicates that binding of δ-cadinene does not induce significant conformational changes in the human MMP-2 structure. This suggests that δ-cadinene maintains the overall structural integrity of the protein after binding, which may be critical for maintaining the functional state of MMP-2. Such structural stability is associated with effective binding without destabilizing the target protein, which could translate into a more specific inhibitory effect. These results are consistent with previous reports that stabilize the conformation of MMP-2 effectively modulate its enzymatic activity without causing deleterious structural disruption [66]. These results support the hypothesis that δ-cadinene may act as an allosteric modulator of MMP-2, interfering binding to the ECM rather than directly inhibiting proteolytic function. This dual binding behavior broadens the therapeutic implications of δ-cadinene. While inhibitor aim to block catalytic zinc [67], δ-cadinene to be able to target both enzymatic and adhesive domains, which may reduce the likelihood of resistance mechanisms or functional compensation. The energy decomposition analysis particularly highlighted key residues such as Tyr131 and Pro133 in the Collagenase I-like region, and Met282 and Phe331 in the Fibronectin type II domain (Figure 9a,c). These residues contributed significantly to the binding energy of the ligand and may serve as important anchors to stabilize the complex. These results suggest that δ-cadinene may function via a non-competitive mechanism by stabilizing the Fibronectin type II domain and hindering access to or anchoring, of the substrate. Our results are consistent with those of Jha et al. [66], who used MD simulations to show that Epigallocatechin-3-gallate (EGCG), a catechin that targets the Fibronectin type II repeats 1 and 3 of MMP-2, binds to key residues and thereby interferes with substrate positioning.

This concordance suggests that binding to non-catalytic domains, such as the Fibronectin type II domain, may represent a viable inhibitory mechanism, supporting the potential of this domain as a novel therapeutic target. It is important to note that in this study MMP-2 activity was not investigated using in vitro biochemical or cellular assays. In contrast, previous experimental studies have confirmed the ability of EGCG to suppress the gelatinolytic activity of MMP-2 in vitro [68]. For example, Cheng et al. [68] reported that EGCG significantly reduced the enzymatic function of MMP-2. These experimental results support the hypothesis proposed by Jhan et al. [66], but also highlight the importance of integrating computational predictions with empirical validation to fully characterize MMPs inhibition by secondary metabolites such as δ-cadinene. To complement our in silico results, a series of in vitro assays were performed to evaluate the biological relevance of δ-cadinene in BC cells. In this study, the TNBC cell line MDA-MB-231 was used, which was selected due to its highly invasive phenotype and increased expression of markers associated with invasion [69,70]. The non-tumorigenic epithelial breast cell line MCF10-A was used as a control to provide a baseline for comparison with malignant cells. The well-characterized aggressive behavior of MDA-MB-231 supports its use as a BC model to investigate the molecular mechanisms targeted by δ-cadinene, thus increasing the translational relevance of the results.

Cytotoxicity was assessed using the MTT assay, which showed that δ-cadinene significantly reduced the viability of MDA-MB-231 cells, with an IC_50_ value of 1.7 ± 0.1 μM at 24 h, 1.7 ± 0.1 μM at 48 h, and a value of 0.6 ± 0.1 μM at 72 h. The experimentally observed cytotoxicity of δ-cadinene at the predicted IC_50_ value (3.5 μM) provides partial validation of the in silico prediction of the PaccMann platform.

Although the expected 50% reduction in MDA-MB-231 cell viability was not achieved, δ-cadinene resulted in a consistent decrease of approximately 19–20% across all time points, indicating that the predicted concentration is within a biologically active range. These models and the actual cellular dynamics, including solubility of the compound, membrane permeability, metabolic degradation, or intracellular accessibility of the target [71]. Nonetheless, the closeness of the experimental response to the predicted value underlines the potential utility of in silico screening tools for lead compounds prioritization, while also highlighting the need for empirical validation in complex biological models such as BC.

In contrast, Paclitaxel—a clinically established chemotherapeutic agent—showed a constant IC_50_ value of 0.3 μM under similar experimental conditions. Although δ-cadinene was less effective than Paclitaxel at earlier time points, its efficacy improved markedly after 72 h, reaching a level of cytotoxic activity that classifies it as a potent agent. According to the IC_50_-based efficacy classification proposed by Krippendorff et al. [72], this development reflects a transition from moderate (IC_50_ between 1 and 10 μM) to potent cytotoxic activity (IC_50_ < 1 μM). This finding suggests that δ-cadinene exerts a delayed but potent antitumor response on BC cells, highlighting its potential value as a therapeutic candidate, particularly when considering its natural origin and potential advantages in toxicity or resistance profiles. Secondary metabolites are structurally diverse natural compounds that are not directly involved in primary metabolic pathways but often exhibit potent biological and pharmacological activities, making them promising therapeutic candidates [56]. The National Cancer Institute Developmental Therapeutics Program (NCI-DTP) has extensively investigated secondary metabolites for their anticancer potential. Initially the compounds are tested with a single high concentration assay at 10 μM for the entire NCI-60 panel of human tumor cell lines [73]. Compounds that reach predefined inhibition thresholds in a minimum number of cell lines are subjected to a more detailed five-concentration screen to determine concentration–response profiles. For example, Irungu et al. [73] evaluated four triperpenoids (EK-2, EK-4, EK-8, and EK-9) using the NCI-60 panel, which showed growth inhibitory effects in selected leukemia lines at concentrations close to 10 μM and IC_50_ values below 5 μM. δ-cadinene showed superior cytotoxic activity against MDA-MB-231 cells, especially when considering that compounds with IC_50_ values ≤ 10 μM are often considered strong leads in NCI-DTP screening. Otherwise, Amaral et al. [74] reported that Cynaropicrin elicited a time- and concentration-dependent effect on MDA-MB-231 cells, with IC_50_ values of 8.05 μM at 48 h and 7.96 μM at 72 h. Their results suggest that prolonged exposure enhances the antiproliferative activity of Cynaropicrin. In our study, δ-cadinene significantly inhibited the cell viability of MDA-MB-231 cells in a time- and concentration-dependent manner, with IC_50_ values decreasing slightly over time. However, δ-cadinene showed a lower IC_50,_ suggesting a better cytotoxic effect than Cynaropicrin. To further evaluate the selectivity profile of δ-cadinene, we compared its cytotoxic activity in non-tumorigenic MCF10-A cells with that of structurally related sesquiterpene lactones previously reported in the literature.

Sotillo et al. [75], investigated in vitro cytotoxicity of natural and synthetic sesquiterpene lactones (Damsin, Ambrosin, Coronopilin, and Dindol-01) against MCF10-A cells. The IC_50_ values reported for Damsin and Ambrosin ranged from 1 to 5 μM, while Coronopilin and Dindol-01 exhibited weaker cytotoxic effects, with IC_50_ values above 10 μM.

In our study, δ-cadinene showed IC_50_ values of 3.2 ± 0.1 μM after 24 h, 2.2 ± 0.1 μM after 48 h and 1.6 ± 0.1 μM after 72 h in MCF10-A cells, placing its activity in the same range of activity as the most active natural compounds reported. However, δ-cadinene showed significantly stronger cytotoxicity against MDA-MB-231 cells, suggesting a more pronounced tumor-selective effect.

On the other hand, in our study, we calculated the selective effect of expressed δ-cadinene using the SI, which is determined by comparing the cytotoxic activity of δ-cadinene against a cancer cell line with its activity against a normal cell line. We have considered the criteria mentioned by Tronina et al. [45]. Thus, the SI values observed in MDA-MB-231 cells indicate that δ-cadinene has higher selectivity against cancer cells than against normal cells, suggesting that δ-cadinene has advantages and potential as a selective compound against BC. These results correlated with morphological assessment by H & E staining, further supporting these observations. The MDA-MB-231 cells showed progressive changes in cell shape, loss of adhesion, and membrane blebbing upon treatment with IC_50_ concentration of δ-cadinene. In contrast, MCF10-A cells retained their epithelial morphology under the same conditions.

The morphological and nuclear changes observed in the present study with δ-cadinene appear to be characteristic of apoptotic cell death [19,42], and have been described in the BC cell line MDA-MB-231 treated with EO of *D. bicolor* containing this metabolite [19]. However, the exact identification of the type of death induced by δ-cadinene requires further studies. These results emphasize the increased efficacy of δ-cadinene and support its further evaluation as a potential anticancer agent. To explore the potential anticancer properties of δ-cadinene against BC cells, we employed a comprehensive strategy combining in silico predictions, cell-based functional assays, and zymographic evaluation of MMP-2 activity. In addition, Transwell invasion assays showed that δ-cadinene (*p* = 0.0439) and Quercetin (*p* = 0.0447), significantly reduced the invasion ability of MDA-MB-231 cells after 24 h, while Paclitaxel (*p* = 0.1378) and 5-FU (*p* = 0.0578) did not produce statistically significant effects under the same conditions. These results confirmed that δ-cadinene significantly reduced the invasive ability of MDA-MB-231 cells. These results suggest that δ-cadinene may affect not only cell viability, but also key mechanisms involved in tumor progression and metastasis. To clarify a possible mechanism underlying these effects, we investigated the activity of MMP-2.

The zymographic analysis showed that δ-cadinene reduced the enzymatic activity of MMP-2 in a time-dependent manner, with a statistically significant reduction observed after 72 h. These results support the hypothesis that δ-cadinene may exert part of its anti-invasive effects via inhibition of MMP-2. These results are consistent with those of Qi et al. [46], where gambogic acid was shown to suppress invasion and MMP-2 activity in MDA-MB-231 cells. Although structurally different, both compounds appear to interfere with ECM-degrading enzymes, further enhancing natural semi-synthetic molecules as relevant scaffolds for anti-metastatic therapies. Importantly, in silico prediction revealed a strong binding affinity of δ-cadinene to MMP-2, particularly within the Fibronectin type II domain, a region implicated in EMC degradation and cell invasion. This interaction suggests a plausible mechanism for the observed anti-invasive effect, δ-cadinene may inhibit the activity of MMP-2 by interfering with substrate recognition and binding, rather than direct catalytic inhibition. Overall, our results suggest that δ-cadinene exerts selective cytotoxic and anti-invasive effects in BC cells, mediated by the stable interaction of δ-cadinene with key domains of MMP-2. The agreement between computational and experimental data emphasizes the potential of δ-cadinene as a candidate for further investigation in the context of metastatic BC.

## 5. Conclusions

Our results show that δ-cadinene exerts a selective cytotoxic and anti-invasive effect on MDA-MB-231 breast cancer cells, while sparing non-tumorigenic MCF10-A cells. In vitro, δ-cadinene significantly reduced cell viability, impaired invasion ability, and induced distinct morphological changes in malignant cells. In silico analyses also revealed strong and stable interactions with both the catalytic and Fibronectin type II domains of MMP-2, suggesting a dual inhibitory mechanism. Overall, these results emphasize the therapeutic relevance of targeting MMP-2–mediated invasion pathways and position δ-cadinene as a promising candidate for the treatment of metastatic BC. Nevertheless, important limitations remain, notably the lack of in vivo validation, which we are currently addressing through ongoing studies, including the development of nanoparticle-based delivery systems to improve the stability, bioavailability, and clinical potential of δ-cadinene.

## Figures and Tables

**Table 1:** Targeted molecular docking analysis of inhibitors on human cell adhesion and migration proteins’ active sites.

Ligand	Protein	ΔG_b_ Estimated (kcal/mol)			Binding Site		Validation	
		Autodock	Vina	Average	Molecular Interactions	Amino Acid Residues	Binding Site	Reference
Quercetin	MMP-9	−7.6	−9.9	−8.7	Hydrogen bonds Alkyl π-Alkyl π-Sigma π-π Stacked	Leu188, Ala189, Val223, His226, Tyr245 Met247	Leu188, Ala189, Glu227, Met247	[36]
	MMP-2	−5.3	−7.1	−6.3	Hydrogen bonds π-Alkyl π-Anion π-π T-shaped	Pro105, Ala108, Phe113, Tyr182, Ala194, Ala 196, Glu412	Arg38, Ile41, Asp45, Gly81, Leu83, Ala84, Ala86, Val117, Glu121, Pro134, Ala136, Ala139	[37]
ADH-1 (Exherin)	N-cadherin	−7.7	−7.3	−7.5	Hydrogen bonds	Val58, Phe60, Val69, Tyr71, Glu83, Asp84	Trp2, Arg23, Arg25, Glu89	[33]
Orientin	ZEB-2	−5.4	−5.1	−5.2	Hydrogen bonds Carbon–Hydrogen bonds π-Sigma π-Alkyl	Leu36, Ser39, Ile40, Leu44	Thr19, Leu20, Ala32, Thr49, Pro51	[35]
Mitoxantrone	CD44	−3.4	−5.1	−4.2	Hydrogen bonds Carbon–Hydrogen bonds π-Sigma	Lys38, Ser45, Arg46, Glu48, Thr111, Ser112, Gln113, Asp167	Arg41, Tyr42, Gln113	[34]

**Table 2:** Binding energy of the molecular docking of δ-cadinene on the active site of human cell adhesion and migration proteins.

Protein	ΔG_b_ Estimated (kcal/mol)			Binding Site	
	Autodock	Vina	Average	Molecular Interactions	Amino Acid Residues
MMP-2	−6.4	−6.3	−6.3	Alkyl π-Alkyl	Pro105, Phe113, Ala196
MMP-9	−6.4	−6.0	−6.2	Alkyl π-Alkyl π-Sigma	Leu188, Val223, His226
N-cadherin	−6.3	−5.6	−5.9	Alkyl	Val69
ZEB-2	−4.9	−4.4	−4.6	Alkyl	Llys50
CD44	−4.8	−4.3	−4.5	π-Alkyl	Tyr42, Tyr114

**Table 3:** Blind molecular docking of δ-cadinene on the human cell adhesion and migration proteins.

Protein	ΔG_b_ Estimated (kcal/mol)	Binding Site	
	Vina	Molecular Interactions	Amino Acid Residues
MMP-2	−7.7	Alkyl π-Alkyl π-Sigma	Met282, Tyr314, Phe331
CD44	−6.4	Alkyl	Leu70, Ile91, Ile96
MMP-9	−6.0	Alkyl π-Alkyl	Leu212, Phe221, Leu222, Phe250
N-cadherin	−6.0	Alkyl π-Alkyl	Pro144, Trp161
ZEB-2	−5.6	Alkyl π-Alkyl	Lys21, Tyr24, Ala25

**Table 4:** IC_50_ values and selective index of δ-cadinene on MDA-MB-231 cells.

	24 h	48 h	72 h
IC_50_ value (μM)	1.7 ± 0.1	1.7 ± 0.1	0.6 ± 0.1
SI	1.8	1.3	2.7

## Data Availability

All data are contained within the article.

## Supplementary Materials

The following supporting information can be downloaded at: https://www.mdpi.com/article/10.3390/cancers17172839/s1. Figure S1: Structural characterization of the N-cadherin protein model. (a) Three-dimensional structure of the constructed N-cadherin model. (b) A plot of the predicted alignment error for the AlphaFold N-cadherin model. The X-axis represents the residue alignment and the Y-axis indicates the expected residues. (c) The Ramachandran plot of the N-cadherin 3D structure was analyzed using the PDBSum platform. Regions of preferred (dark), allowed (light), and disallowed (white) φ/ψ angles are indicated. (d) A Z-score plot was generated using the ProSA-web server. The red arrow indicates the Z-score of the N-cadherin protein. (e) The global energy profile is based on ProSA-web validation. (f) Error quantification using ERRAT. Gray bars represent error-free residues. Yellow bars indicate residues with errors in the 95-99% range. Red indicates residues with errors greater than 99%. Figure S2: Structural models of MMP-2 obtained from the AlphaFold Protein Structure Database: (a) without Zn^2+^, (b) with Zn^2+^ (yellow), (c–d) δ-cadinene docking (red) in proteins without and (green) with Zn^2+^, (e) superposition of both structures, and (f) enlarged view of the binding site showing ligands in similar positions and Zn^2+^.

## Abbreviations

The following abbreviations are used in this manuscript:

BC	Breast cancer
DMEM	Dulbecco’s modified Eagle’s medium
DMSO	Dimethyl sulfoxide
ECM	Extracellular matrix
EMT	Epithelial-to-mesenchymal transition
EO	Essential oil
EOs	Essential oils
FBS	Fetal bovine serum
MD	Molecular docking
MDS	Molecular dynamics simulations
MMPs	Matrix metalloproteinases
Rg	Radius of gyration
RMSD	Root mean square deviation
RMSF	Root mean square fluctuation
SI	Selectivity index
SEM	Standard error of mean
