# Supplementary material for: In Silico and In Vitro Evaluation of δ-cadinene from Decatropis bicolor as a Selective Inhibitor of Human Cell Adhesion and Invasion Proteins
Source: Cancers (Basel). 2025 Aug 29;17(17):2839. doi: 10.3390/cancers17172839 (PMC12427431; doi:10.3390/cancers17172839)
Supplement: Supplementary file 1 [file cancers-17-02839-s001.zip › cancers-3797605-supplementary.pdf]

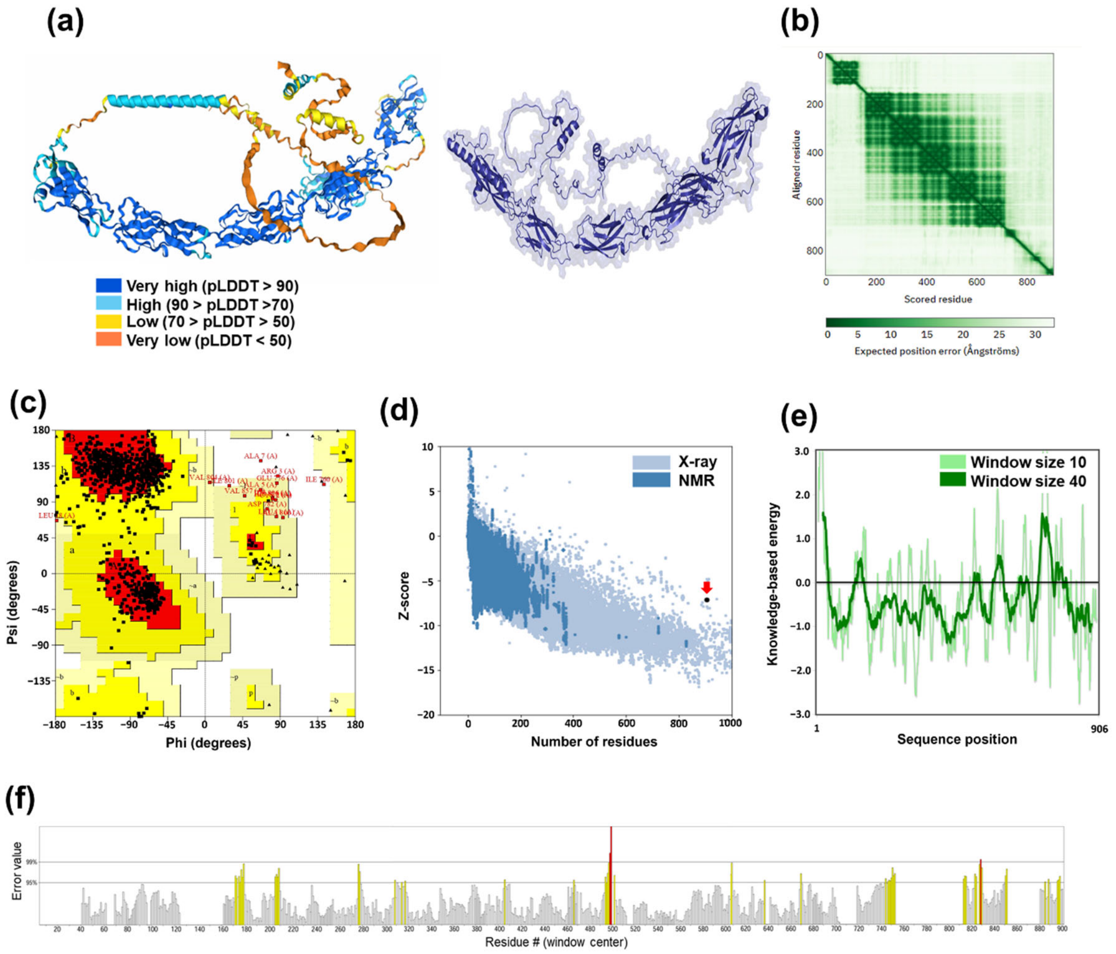


**Supplementary Figure S1.** Structural characterization of the N-cadherin protein model. (a) Three-dimensional structure of the constructed N-cadherin model. (b) A plot of the predicted alignment error for the AlphaFold3 N-cadherin model. The X-axis represents the residue alignment and the Y-axis indicates the expected residues. (c) The Ramachandran plot of the N-cadherin 3D structure was analyzed using the PDBSum platform. Regions of preferred (dark), allowed (light), and disallowed (white) φ/ψ angles are indicated. (d) A Z-score plot was generated using the ProSA-web server. The red arrow indicates the Z-score of the N-cadherin protein. (e) The global energy profile is based on ProSA-web validation. (f) Error quantification using ERRAT. Gray bars represent error-free residues. Yellow bars indicate residues with errors in the 95-99% range. Red indicates residues with errors greater than 99%.

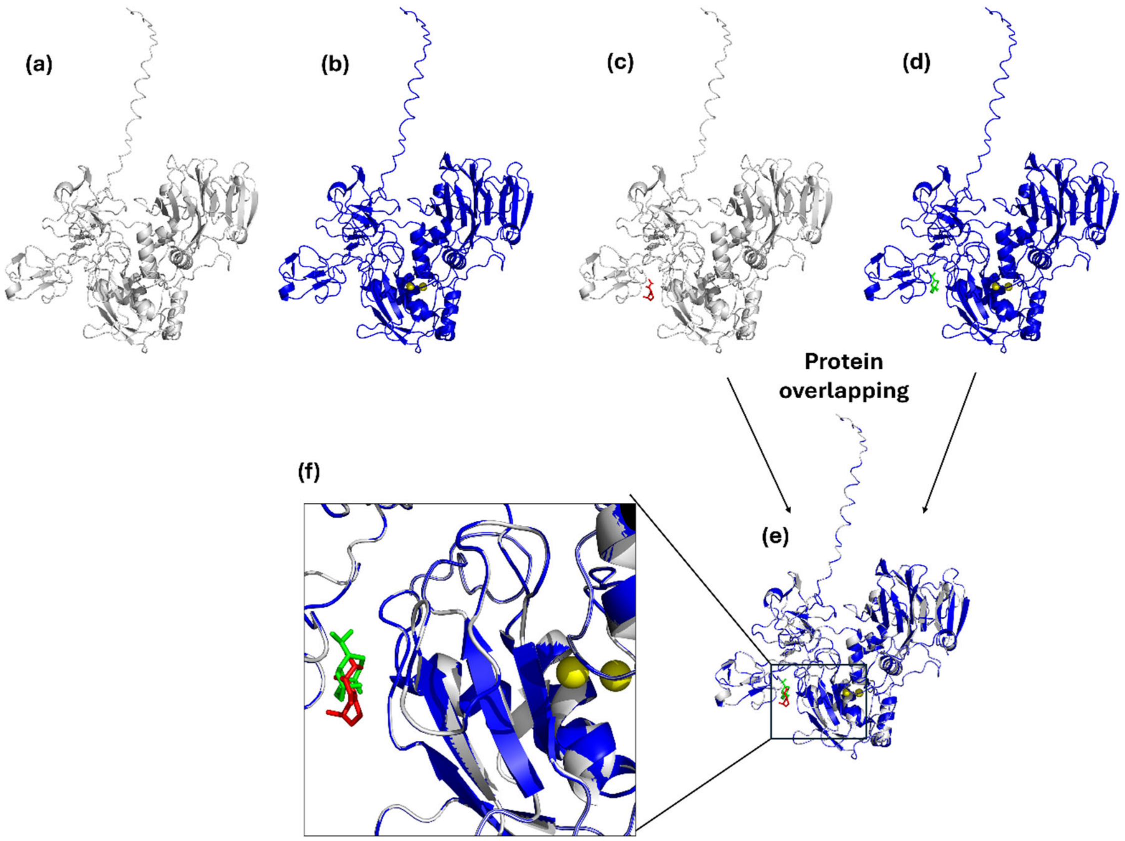


**Supplementary Figure S2.** Structural models of MMP-2 obtained from the AlphaFold Protein Structure Database: (a) without $Zn^{2+}$, (b) with $Zn^{2+}$ (yellow), (c–d) δ-cadinene docking (red) in proteins without and (green) with $Zn^{2+}$, (e) superposition of both structures, and (f) enlarged view of the binding site showing ligands in similar positions and $Zn^{2+}$.
